# Kinetic Profiling in One-Step Digital Immunoassays Enables Multiplex Quantification across an Ultrabroad Dynamic Range

**DOI:** 10.1021/jacs.5c17838

**Published:** 2026-01-27

**Authors:** Abtin Saateh, Rojina Allamehnejad, Wenhong Yang, Yen-Cheng Liu, Genrich V. Tolstonog, Hatice Altug

**Affiliations:** † Institute of Bioengineering, 27218 École Polytechnique Fédérale de Lausanne (EPFL), Lausanne CH-1015, Switzerland; ‡ Department of Otolaryngology-Head and Neck Surgery, 30635 Lausanne University Hospital and University of Lausanne, Lausanne 1011, Switzerland; § AGORA Cancer Research Center, Lausanne 1005, Switzerland

## Abstract

In one-step sandwich immunoassays, where all binding components coexist in solution, excessive analyte levels can inhibit sandwich complex formation by competing with labeled detection antibodies, producing the well-known “hook effect.” Here we establish a kinetic framework that resolves this ambiguity by analyzing time-resolved single-particle plasmonic signals. Using gold nanohole arrays with nanoparticle reporters, we continuously track individual binding events and fit their response-time profiles to both mass-transport- and reaction-limited models. Comparison of fit residuals identifies the dominant mechanism in each concentration regime, revealing the kinetic transition that gives rise to the hook effect and converting it to a quantitative feature. The digital framework also classifies and mathematically decouples distinct types of cross-reactivity in multiplexed assays, minimizing off-target interference. Applied to multiplexed detection of cytokines and C-reactive protein in unprocessed human serum, our approach enables simultaneous quantification of low- and high-abundance biomarkers, ranging in total over 9 orders of magnitude, without sample splitting or analyte-specific dilution. This mechanistic strategy establishes a generalizable paradigm for kinetic, cross-reactivity-aware biosensing.

## Graphical abstract

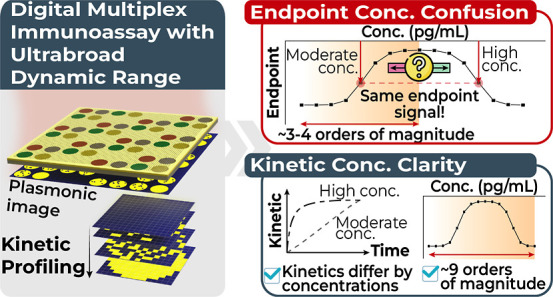

## 1 Introduction

Biosensing technologies are critical to clinical diagnostics and biomedical research, enabling the sensitive, rapid, and accurate detection of biomarkers across a range of disease states. Traditional assays such as enzyme-linked immunosorbent assays (ELISAs) offer high specificity but are inherently limited to single-analyte detection per run.^1,2^ This single-plex format is increasingly insufficient in the context of complex diseases, where no single biomarker can fully capture the underlying biology.^3^

Multiplexed biomarker panels provide richer diagnostic information by offering a comprehensive disease picture.^4,5^ For example, cytokine profiling can reveal immune dysregulation in inflammatory diseases or response to immunotherapy.^6–8^ Biosensors offering multiplexed detection improves clinical decision-making,^9,10^ accelerates assay workflows, and minimizes the sample volume required, an essential consideration in limited patient samples.^11,12^

Several technologies such as microarrays, bead-based assays, and spatially encoded sensors have been developed for high-throughput parallel measurements.^13^ However, a shared limitation across current diagnostic systems is their restricted dynamic range, typically spanning only 3–4 logarithmic orders of magnitude.^4^ This constraint poses a significant challenge when biomarkers of clinical interest appear at vastly different concentrations in a given sample, for example, cytokines in the pg/mL range^14,15^ versus acute-phase proteins like CRP in the μg/mL range.^16^ Among commercially available multiplex solutions, the Luminex technology is widely adopted. When confronted with high-abundance target biomarkers like CRP, it requires extensive sample dilution to avoid signal saturation. But the inclusion of a dilution step drives the low-abundance targets present at pg/mL levels, such as cytokines, below the detection limit of the sensor. As a result, they suffer from the capability of simultaneously quantifying both high and low-concentration biomarkers in a single run without sample splitting. In addition, dilution introduces nonlinear effects that compromise quantification accuracy.^17^ Recent efforts to address dynamic range limitations have focused on tuning antibody concentrations either by increasing free antibody levels to shift the binding equilibrium and elevate the upper quantifiable limit^18,19^ or by raising tagged antibody concentrations to enhance sensitivity^19^ but at the expense of increased assay complexity.

Beyond limitations in the dynamic range, the performance of multiplexed biosensors can be further constrained by the readout strategy. Conventional platforms predominantly rely on ensemble averaging and end point detection, where a bulk signal from a large population is probed at a single time point.^20^ For instance, systems such as Luminex provide a snapshot of accumulated fluorescence signal intensity from all the beads without monitoring the real-time binding behavior of analytes. Ensemble measurements struggle with limited sensitivity, particularly at low analyte concentrations, whereas the end point readout misses to capture the kinetic data, such as saturation profiles, or temporal trends, which can be highly informative to distinguish between different analyte concentrations. A striking case concerns the hook effect, where excessively high analyte levels lead to paradoxically reduced signal, leading to false-negatives.^21,22^ Previous studies have shown that by transitioning to dynamic monitoring in conventional lateral flow assays, the hook effect can be harnessed as a diagnostic feature, where the full signal evolution can be captured and interpreted.^23,24^

To achieve low limits of detections and high sensitivity, an emerging direction in optical sensing is “digital resolution” read-out.^25,26^ Unlike traditional refractometric biosensors, such as surface plasmon resonance (SPR), which monitor an averaged resonance shift caused by collective analyte accumulation,^27^ digital affinity sensors enable direct counting of individual target molecules and observation of single binding events. As a result, a digital biosensor can provide a signal even at very low analyte concentrations. For example, Tao and colleagues coupled gold nanoparticles (AuNPs) to an SPR imaging system, where near-field plasmonic interactions generated high-contrast diffraction patterns for each AuNP^28^ to enable a time-resolved digital immunoassay with a detection limit of 2.8 pg/mL.^29^ Zijlstra group have explored nanoparticle-on-film systems for single-molecule digital detection using dark-field microscopy.^30^ Using a nanophotonic approach, Belushkin et al. developed a portable digital biosensor based on gold nanohole arrays (AuNHAs) and nanoparticles with detection limits in the 20–30 pg/mL range and evaluated several biomarkers in single-plex format within artificial complex matrices (e.g., 7% BSA) for point-of-care applications.^31,32^ In parallel, Cunningham and co-workers used dielectric nanostructures supporting guided-mode resonances for digital immunoassays with improved stability and single-particle resolution.^33,34^ Despite the recent progress, digital nanophotonics biosensors, which are inherently suited for multiplexed and real-time signal acquisition and miniaturization, are still in their infancy, with dynamic ranges typically limited to 3–4 orders of magnitude.

Despite extensive efforts to extend the dynamic range through antibody or reagent tuning, the fundamental kinetic transition between affinity and competition regimes remains unresolved. Conventional ensemble assays measure only end point responses averaged over large populations, concealing this mechanistic shift. Existing digital biosensors, while achieving single-particle sensitivity, are typically operated at end points or under conditions that deliberately avoid the hook regime. Capturing binding dynamics in real time at the single-particle level, however, can expose these distinct kinetic signatures and fundamentally redefine how the hook effect is interpreted.

In this work, we introduce a nanoplasmonic digital microarray, PLASM-ART (Plasmonic Large-dynamic-range Analysis Sensor for Multiplexed Assay and Rapid Tracking), that uniquely combines kinetic read-out with digital single-particle detection, enabling mechanistic discrimination of binding behavior even when end point signals converge. Remarkably, this synergistic integration, which allows the extraction of kinetic signatures from individual AuNP binding events in a spatially and temporally resolved manner, enabled us to dramatically extend the quantifiable dynamic range across 9 orders of magnitude in total from fg/mL to μg/mL without the need for analyte-specific dilution or sample splitting. The ability to access kinetic signatures provided simultaneous detection of both low-abundance cytokines and high-abundance acute-phase proteins within a single assay run. We demonstrated the microarray operation for the parallel detection of four selected disease biomarkers (IL-2, IL-6, IFN-γ, and CRP) at high specificity and without crosstalk. Using less than 10 μL of unprocessed human serum, the nanoplasmonic digital microarray enables direct analysis of human serum while avoiding quantification errors introduced by nonlinear dilution effects. To overcome a persistent and often-overlooked limitation of cross-reactivity in multiplexed clinical assays, we implemented a correction strategy that minimizes cross-reactivity effect without compromising true analyte detection. Quantification is achieved within 15 min, offering speed, sensitivity, and precision suitable for point-of-care deployment.

## 2 Results and Discussion

### 2.1 System Concept and Detection Principle

As illustrated in Figure 1A, the nanoplasmonic microarray sensor consists of a periodic array of nanoholes patterned in a thin gold film functionalized with spatially defined antibody spots. Each region serves as a discrete sensing zone for multiplexed detection of disease biomarkers. In periodic gold nanohole array (AuNHA) structures, excitation of localized plasmons that couple with propagating surface plasmons enables light to transmit through a gold film thicker than the skin depth and through subwavelength apertures, a phenomenon known as extraordinary optical transmission EOT.^35,36^ Upon narrowband illumination, the EOT signal intensity supported by the AuNHA is captured as an image in real-time with a CMOS camera. Digital detection is achieved through signal generation by the individual AuNPs brought into the evanescent field of the AuNHA surface upon analyte binding (Figure 1A, right). Upon the presence of the analyte, functionalized AuNPs bind selectively on the NHA interface. Given that the EOT phenomenon is extremely sensitive to the changes occurring near the surface, the bound gold NPs on the substrate perturb the plasmonic modes of the AuNHA and locally suppress the EOT intensity at the detection wavelength. These localized signal suppression events are observed as high-contrast dark pixels on the CMOS imager for each binding event and enable quantifiable readouts by converting molecular recognition into spatially resolved digital data in the form of kinetic image stacks. Unlike ensemble-averaged peak-shift-based refractometric sensors requiring the accumulation of a large number of analytes, this digital method is inherently more sensitive and resistant to the baseline drift and optical noise.

**Figure 1:**
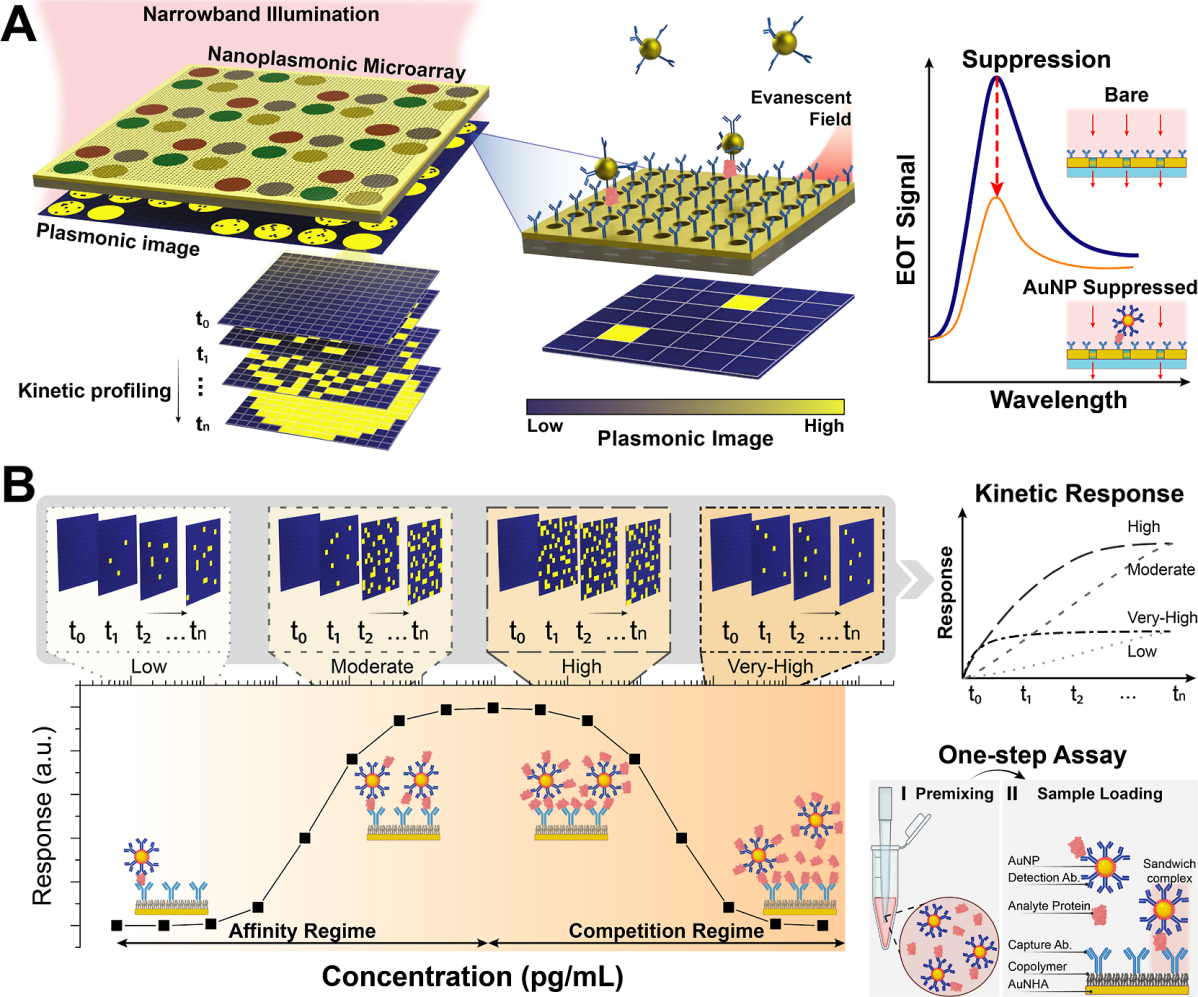
A digital nanoplasmonic microarray biosensor, PLASM-ART, based on AuNHAs coupled with AuNPs for real-time, quantitative biomarker detection. (A) Overview of the nanoplasmonic imaging biosensor architecture and sensing mechanism. A nanohole-patterned gold film functionalized with antibody microarrays enables spatially multiplexed detection under narrowband illumination. Binding of AuNP-analyte complexes to capture antibodies on the surface of the AuNHA substrate perturbs the extraordinary optical transmission (EOT) signal within the evanescent field, generating discrete dark pixels that are captured in real time by a CMOS camera. This integrated panel illustrates the spatial readout, digital particle-level detection, and the mechanistic basis of EOT-based signal generation on the AuNHA surface. (B) Assay workflow, competitive mechanism, and resulting kinetic behavior. The assay operates in a one-step format in which the sample is premixed with AuNP-detection antibody conjugates before loading onto the AuNHA chip. At low to moderate analyte concentrations, complex formation leads to gradual, rising kinetic profiles over time, defining the affinity regime of the calibration curve. At higher concentrations, excess free analyte suppresses complex formation, resulting in fewer binding events and fast kinetics that plateau rapidly, producing the descending, competition regime of the bell-shaped curve. These concentration-dependent kinetic signatures illustrate the influence of the competition on the assay response. The corresponding kinetic response-time plot extracted from time-resolved images consolidates these behaviors.

The detection exploits a streamlined one-step sandwich immunoassay format, as shown in Figure 1B (bottom right). Biological samples, typically serum, are first premixed with AuNPs conjugated to detection antibodies, enabling analytes to form immune complexes in solution. Upon introduction to the AuNHA chip, these complexes are then selectively captured by the corresponding capture antibodies, forming a complete sandwich structure that anchors AuNPs to the sensor surface.

Crucially, the continuous acquisition of image stacks provides temporally and spatially resolved digital signals with distinct kinetic behaviors, depending on the analyte concentration. The analysis of these kinetic signatures resulting from the underlying binding dynamics facilitates the discrimination of different concentration-dependent regimes and overcomes the ambiguity inherent to measurements based solely on end-point readouts. As shown in Figure 1B, kinetic insets corresponding to each concentration range depict the time-resolved evolution of plasmonic signal suppression, while the top-right panel shows the corresponding kinetics profiles as response-time plots across different concentration ranges. For example, at low analyte concentrations, the kinetic signal onset is delayed and progresses gradually as the sandwich complexes slowly accumulate. Moderate concentrations yield a rapid signal rise and sustained intensity, characteristic of efficient binding kinetics, whereas high to very high concentrations exhibit a fast initial signal appearance followed by a suppressed response, indicative of the competition regime where excess free analyte impairs ternary complex formation. This temporal profiling empowers our sensor to distinguish between similar end point signals that would otherwise be confounded by the hook effect.

In the calibration curve, these distinct kinetic behaviors give rise to a biphasic, bell-shaped dose–response curve with two detection regimes: an affinity regime at low to moderate concentrations and a competition-limited at high to very high concentrations. In the affinity regime, sandwich-complex formation follows predictable binding kinetics, leading to a gradual but sustained increase in the level of local signal suppression. In contrast, at high to very high concentrations, excess analyte saturates binding sites on both the surface and AuNPs, inhibiting ternary complex formation, a phenomenon known as the hook effect, and resulting in signal attenuation despite increased analyte abundance. Together, the kinetic readouts and their corresponding end-point responses enable a dramatic expansion of the dynamic range for quantitative detection, spanning over 9 orders of magnitude in total for the analyte concentration.

### 2.2 Analytical Performance in Buffer

To enable quantitative biosensing, we first fabricated large-area AuNHAs and established a microarray chip architecture. As shown in Figure 2A, AuNHAs were uniformly manufactured at the wafer scale via deep ultraviolet lithography (DUVL), yielding over 50 individual chips per 4 in. glass wafer, thereby enabling cost-effective, disposable chip per measurement. Each nanohole array featured a periodic pattern of 200 nm diameter holes with 600 nm center-to-center spacing in a 100 nm thick gold film deposited on a glass substrate with a 5 nm titanium adhesion layer. The chips were diced into 1 × 1 cm^2^ units with highly uniform EOT spectra across the sensor. For large-area plasmonic image acquisition, a collimated LED source was used to illuminate the array from above and transmission images were recorded using a monochrome CMOS camera. Imaging was performed using a, 3D-printed microscope platform based on the OpenFlexure design,^37^ which enables precise control of the sample stage in *x*, *y*, *z*, directions with submicron resolution.

**Figure 2:**
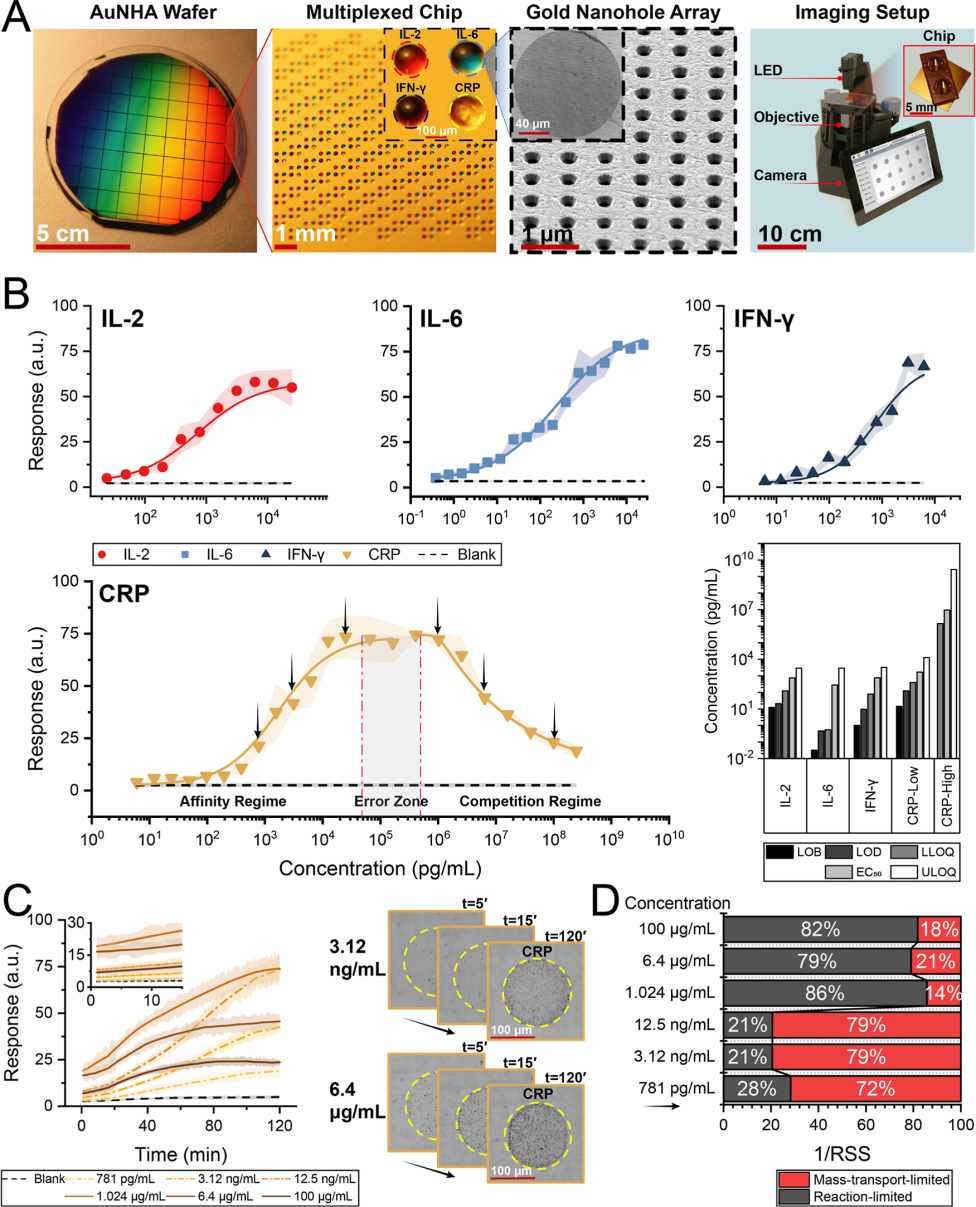
Kinetic detection of disease biomarkers using a digital nanoplasmonic microarray biosensor. (A) Left to right: optical image of a 4 in. AuNHA wafer consisting of over 50 1 × 1 cm^2^ chips fabricated by deep UV lithography; zoomed-in view of a multiplexed chip with antibody microarrays on AuNHA, with inset highlighting a microarray pattern (colored with food dye for illustration); tilted SEM image showing uniform AuNHA geometry, with inset showing a single antibody microarray spot; and an optical image of a custom-built optical reader based on a modified 3D-printed OpenFlexure microscope adapted for signal acquisition from the nanoplasmonic chip. (B) Calibration curves for IL-2, IL-6, IFN-γ, and CRP in buffer under multiplexed conditions. Sensor responses followed sigmoidal logistic fits with high correlation (*R* ^2^ > 0.96). CRP exhibited a biphasic response, with an error zone separating affinity and competition regimes; the boundaries of this zone are marked with dot-dash vertical lines, corresponding respectively to the ULOQ of the affinity regime and the LLOQ of the competition regime, where each regime was fitted independently. Key assay performance metrics including limit of blank (LOB), limit of detection (LOD), lower limit of quantification (LLOQ), half-maximal effective concentration (EC_50_), and upper limit of quantification (ULOQ) are summarized in the adjacent bar plot. (C) Left: time-resolved kinetic response analysis of CRP across six representative concentrations (indicated with black arrows in panel A), revealing distinct kinetic profiles in affinity and competition regimes; inset highlights early binding events (≤15 min). Right: image sequences of CRP binding at 3.12 ng/mL and 6.4 μg/mL at three selected time points over the 120 min interval. (D) Binding regime analysis using inverse relative normalized residuals (1/RSS) from mass transport-limited and reaction-limited kinetic fits. Bars indicate the relative residuals of the two fits to each kinetic profile, normalized to reveal which model better describes the data. Low concentrations align with mass transport-limited behavior, whereas high concentrations align with reaction-limited behavior.

The chips were biofunctionalized in two steps. First, the AuNHA surface was chemically modified with a block copolymer containing activated NHS groups to enable biomolecule attachment. Then, all capture antibodies were immobilized onto the microarray chip by spot functionalization using a noncontact piezoelectric dispensing system. Separately, AuNPs were functionalized with the corresponding detection antibodies for each target (see Methods for full details). The morphology and optical properties of the AuNPs and antibody-conjugated AuNPs are shown in Figures S1–S3, with corresponding quantitative values summarized in Table S1.

To evaluate the quantitative performance of our sensor, we next conducted multiplexed calibration experiments targeting IL-2, IL-6, IFN-γ, and CRP across a broad dynamic range using buffered standards. The binding buffer used in these assays was optimized through a Taguchi screening strategy to maximize specific signal and reduce variability, as detailed in Figures S4 and S5. To perform these biosensing experiments, a detection solution containing all four AuNP-detection antibody conjugates was used in each assay. The only variable across individual calibration runs was the concentration of the spiked analyte, ensuring that the sensor performance was evaluated under realistic multiplexed conditions. The concentrations of IL-2, IL-6, and IFN-γ ranged from low picogram levels up to 25 ng/mL, while CRP extended from the same starting range to 250 μg/mL. Each condition was measured in triplicate to assess reproducibility. As a control, we used blank samples containing all detection AuNPs but lacking analytes on the microarray chip functionalized with all four capture antibodies.

The dose-dependent responses of IL-2, IL-6, and IFN-γ, shown in Figure 2B, yielded characteristic sigmoidal S-shaped calibration curves with high correlation coefficients (*R* ^2^ = 0.97, 0.98, and 0.96, respectively). The sensor achieved subpicogram sensitivity, with limit of blank (LOB) of 35 fg/mL and limit of detection (LOD) as low as ∼460 fg/mL for IL-6 and dynamic ranges extending up to the ng/mL level (calculation details in Supporting Information).

The dose-dependent response of CRP presented a biphasic curve spanning over 8 orders of magnitude, from 100 pg/mL to 100 μg/mL. It consists of S and reversed S-shaped regions at low and high concentrations, corresponding to the affinity and competition regimes, respectively. A central error zone emerged at their interface as a concentration range in which the analytical signal transitions between two mechanistically distinct regimes of binding behavior. Here, reliable quantification becomes compromised due to signal nonlinearity and high sensitivity to small fluctuations. We quantified the error zone as the range between the upper limit of quantification (ULOQ) of the affinity regime and the lower limit of quantification (LLOQ) of the competition regime. As a result, this zone, spanning approximately 1 order of magnitude in this example, was excluded from quantification. Key analytical parameters, including LOB, LOD, LLOQ, half-maximal effective concentration (EC_50_), and ULOQ, are summarized in the accompanying bar graph (Figure 2B, bottom right), underscoring the assay’s broad dynamic range and quantitative outcome for all four biomarkers.

Real-time signal traces for CRP detection provided critical insights into binding kinetics across concentration ranges (Figure 2C, left). Six representative concentrations were selected: three from the affinity regime and three from the competition regime. Within each regime, a pair of concentrations with similar end point responses were chosen to highlight the differences in their kinetic profiles that would be otherwise invisible in static measurements. The main plot displays the sensor signal over time, while the inset above zooms into early time points, demonstrating that meaningful detection can be achieved in less than 15 min. Even at the early stages, the binding signal from analyte-containing samples clearly diverged from the blank response (black dashed line).

To further illustrate kinetic differences, image stacks were acquired from the CRP capture spot on chips exposed to two representative concentrations, one from each regime, and compared side-by-side at early (*t* = 5 min) and late (*t* = 120 min) time points (Figure 2C, right). While each concentration was tested on a separate chip, the images at each concentration show the same spatial region (CRP spot) monitored over time. In the affinity regime (3.12 ng/mL), signal accumulation was initially slow, with a gradual intensity increase over time. In contrast, the competition regime (6.4 μg/mL) already exhibited a strong signal at early stages, which plateaued quickly as the binding sites became saturated. These temporal intensity patterns visually reinforce the transition between two regimes across the biphasic curve, predicted by the competitive model in Figure S6.

In the affinity regime, signal accumulation is primarily governed by the diffusion of AuNP-analyte complexes toward the sensing surface. Since the AuNPs are relatively large and premixed with the analytes before chip application, mass transport (diffusion through the stagnant layer) remains the dominant limitation during binding. In contrast, in the competition regime, the sample contains an excess of free analytes alongside analyte-AuNP complexes. Because free analytes are significantly smaller than the AuNPs, they diffuse orders of magnitude faster, altering the kinetics: binding becomes reaction-limited, governed by the probability of forming stable ternary complexes on the surface rather than diffusion alone. This regime transition is dictated by the inherent diffusion contrast between AuNP-analyte complexes and the free proteins. Thus, it is general and not biomarker-specific, enabling regime identification by using the same kinetic framework for different targets.

To quantitatively capture the differences in binding dynamics in two regimes, we fitted the measured kinetic profiles to idealized mass transport-limited and reaction-limited models and compared their quality using inverse relative normalized residuals (1/RSS), where higher values indicate a better fit. In Figure 2D, each bar reports the relative residuals of the two different models fitted individually to the same kinetic profile. The residuals from the fits were normalized to a common 100% basis to enable comparison and highlight which model better describes the data. Values close to 1 indicate strong agreement with the corresponding model. Concentrations within the error-zone were excluded because their kinetic profiles there are nearly indistinguishable. As illustrated in Figure 2D, the kinetic profiles in the affinity regime aligned more closely with mass transport-limited behavior, whereas the profiles in the competition regime were better described by reaction-limited behavior. This analysis enables reliable regime discrimination even when end point signals are comparable.

Although demonstrated here using a nanoplasmonic digital platform, this kinetic framework is broadly applicable to any surface-based one-step assay governed by competing binding equilibria, including electrochemical, field-effect-, and fluorescence-based systems.

### 2.3 Analytical Characterization in Human Blood Serum

Next, we demonstrated the applicability of PLASM-ART for biomarker detection in human blood serum, a complex matrix where both biological cross-reactivity and matrix interference can significantly impact the specificity and quantitative accuracy. Although cross-reactivity is a known concern, only a few studies have systematically examined its extent and implications in multiplex immunoassays.^38–40^ To transition from buffer to serum, we considered analysis of two critical aspects: cross-reactivity between assay reagents (capture/detection antibodies and target proteins) and matrix interference from serum components for ensuring quantitative fidelity in multiplexed detection.

To assess specificity under serum conditions, we first investigated the reagent-driven cross-reactivity in the absence of complex matrix interference. Each antibody–analyte pair was tested individually to isolate the contributions of noncognate interactions. These included three mechanistic sources: nonspecific binding of detection antibodies to capture zones (dAb-cAb), unintended binding of detection antibodies to nontarget analytes in solution (dAb-analyte), and binding of nontarget analytes to capture antibodies (analyte-cAb) (see Figure S7 for mechanism and calculations). As detailed in the SI (Figure S8), the majority of extracted cross-reactivity coefficients remained below 5%, confirming minimal off-target interactions in the buffer.

Initial testing in human serum occasionally revealed an elevated background at the IL-2 capture site, despite the absence of IL-2 in the sample (Figure S9B). This effect was not observed in buffer or bovine serum and was effectively mitigated through a pretreatment strategy detailed in the SI (Figure S9E). Following this adjustment, all capture zones displayed clear analyte-specific responses (Figure 3A first panel). Under this unspiked analyte condition, only the CRP capture spot showed a significant signal, reflecting the presence of endogenous CRP in human serum. In panels two to four of Figure 3A, IL-2, IL-6, and IFN-γ were individually spiked at 10 ng/mL, resulting in localized signal increases at their respective capture zones, while the CRP spot continued to show a baseline signal due to endogenous content, despite no added CRP. In panel five of Figure 3A, CRP was spiked at 40 μg/mL, producing a biphasic response, with signal suppression, consistent with a hook effect previously observed in buffer. Full multiplexed spiking of all four biomarkers (Figure 3B) further confirmed that analyte-specific responses dominated the signal distribution.

**Figure 3:**
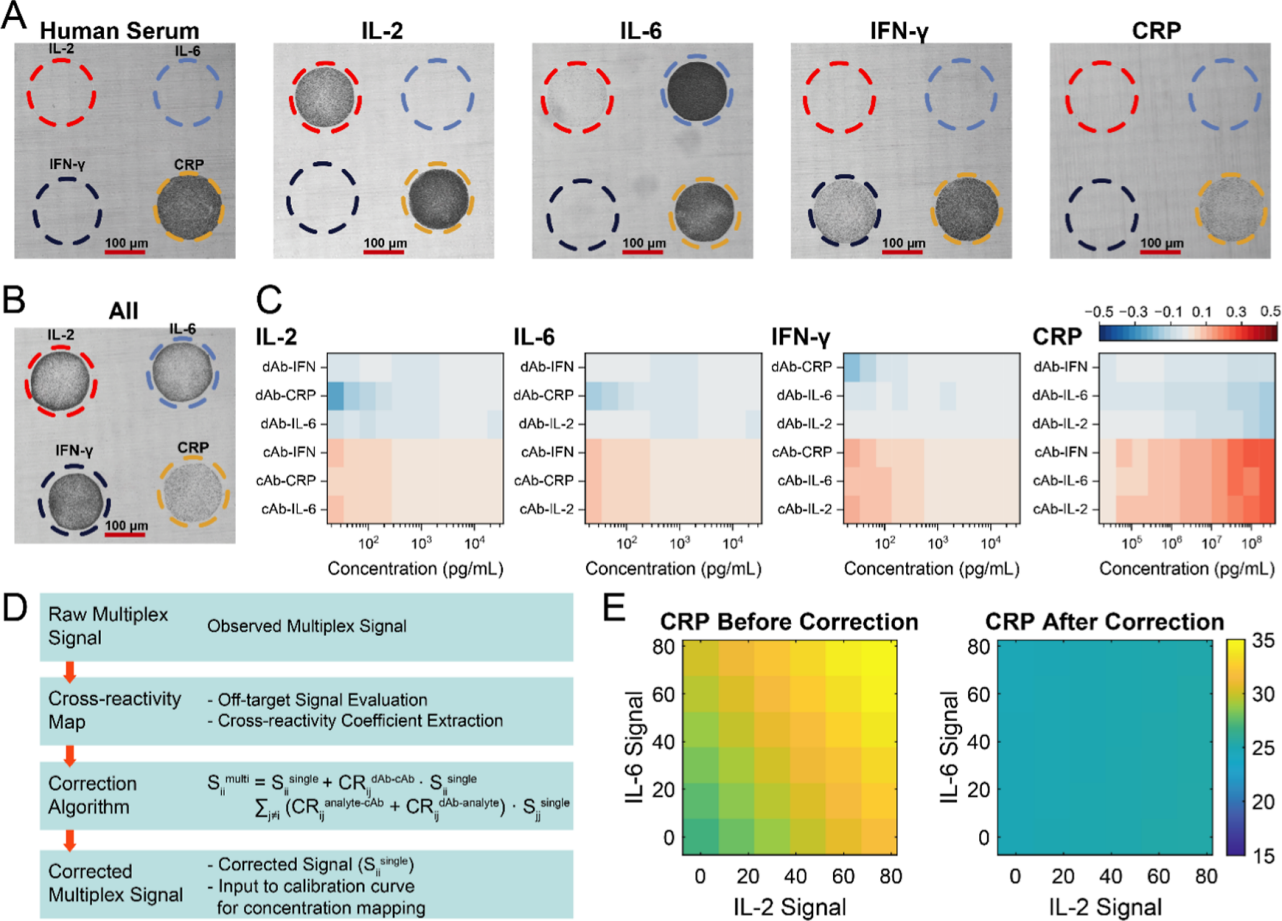
Analytical characterization and cross-reactivity correction in human serum. (A) Multiplexed signal response in unspiked human serum. The result shows a strong CRP signal due to endogenous levels in the sample. Upon individual spiking of IL-2, IL-6, or IFN-γ (10 ng/mL each), signal increases are observed at their respective capture zones, while the CRP signal remains detectable. In the CRP-spiked sample (40 μg/mL), the CRP signal decreases, consistent with the hook effect at high concentrations. (B) Multiplexed response in serum containing all four spiked biomarkers of IL-2, IL-6, and IFN-γ at 10 ng/mL and CRP at 40 μg/mL. (C) Extracted cross-reactivity coefficients in serum for each biomarker channel, shown as heatmaps across a wide concentration range. Coefficients correspond to analyte binding to off-target capture antibodies (cAb-analyte) and detection antibody binding to noncognate analytes (dAb-analyte). Most values remained below 12%, with higher off-target effects observed at elevated CRP concentrations. (D) Schematic of the signal correction workflow. Observed multiplexed signals are corrected using extracted cross-reactivity coefficients, incorporating all three interaction types as described in the correction formula. (E) Correction framework for CRP. Before correction, CRP signals were confounded by increasing IL-2 and IL-6 signals. After applying the algorithm, CRP values became independent of coanalyte interference, recovering single-analyte signal equivalence across the full range.

To quantify cross-reactivity under multiplexed serum conditions, observed signals were decomposed by using the previously defined interaction model. Cross-reactivity coefficients were computed across a wide concentration range and visualized as heatmaps for each biomarker (Figure 3C). These maps reveal the magnitude of off-target contributions from each noncognate interaction pathway.

Most coefficients remained below 12%, showing the overall specificity of the assay. For IL-2, IL-6, and IFN-γ, cross-reactivity effects were minimal across the full concentration range, as evidenced by the heatmap colors fading toward white at higher analyte levels. This indicates that increasing concentrations of these cytokines do not introduce notable off-target interactions, highlighting their biochemical orthogonality under the assay conditions.

In contrast, CRP displayed a distinct cross-reactivity profile with elevated coefficients at high concentrations. Notably, positive values in the cAb-IL-2 and cAb-IL-6 rows indicate that CRP binds noncognate capture antibodies. Simultaneously, negative coefficients in the dAb-IL-2 and dAb-IL-6 rows reflect signal suppression due to competitive inhibition, where CRP binds detection antibodies without forming productive sandwich complexes. This dual behavior, signal elevation through capture antibody binding and suppression through detection antibody competition, highlights the unique interference potential of CRP in multiplexed assays. Importantly, the overall cross-reactivity pattern in serum closely mirrored that observed in the buffer, confirming that matrix effects did not substantially alter reagent specificity and that off-target interactions are predominantly governed by intrinsic binding properties of the assay components.

To correct for these residual effects, we implemented a signal correction algorithm accounting for all three sources of cross-reactivity. Each interaction contributes for a defined fraction of the total signal at a given capture site, and their intensities were quantified as cross-reactivity coefficients during the assay characterization phase. Utilizing these coefficients, we were able to analytically subtract the predicted off-target contributions from each multiplexed measurement. The full correction model is detailed in Figure 3D and Supporting Information Section 6.

This correction was applied to all multiplexed serum measurements (Figure S10). Figure 3E highlights CRP as a representative example before and after correction. In the uncorrected data, biomarker signals were not independent but influenced by the presence of coanalytes, reflecting interference effects arising from cross-reactivity and overlapping detection mechanisms. For instance, CRP signals were visibly perturbed by high IL-2 and IL-6 levels, resulting in increased or suppressed responses across the measurement space. After applying the correction algorithm, the CRP signal became invariant to coanalyte concentrations and converged toward its expected single-analyte profile. This case highlights the correction framework’s ability to disentangle overlapping signal contributions to restore analytical specificity.

To evaluate the broader applicability of our approach, correction was extended to all biomarker channels and tested across the full input space of coanalyte combinations. Prior to correction, IL-2, IFN-γ, and CRP signals were distorted by interference from highly abundant or strongly binding coanalytes, most notably in regions of elevated CRP, IL-6, or IFN-γ concentration (Figure S10). In contrast, the IL-6 signal was largely insensitive to interference, exhibiting the lowest degree of distortion across the multiplex input space. Upon correction, signals for all targets became decoupled from off-target contributions, yielding response surfaces that were flat across orthogonal axes and aligned closely with the single-analyte calibration. This strategy restores specificity while enhancing reliability in multiplexed serum measurements without requiring serial measurements or physical separation of targets.

### 2.4 Clinical Matrix Performance

To assess the clinical applicability of our platform, we evaluated its performance for multiplexed detection by spiking four inflammatory biomarkers in human blood serum. The panel in Figure 4A presents dose–response calibration curves for each biomarker measured directly in human serum. All calibration curves were fitted using 5-PL models, yielding high correlation coefficients (*R* ^2^ = 0.98 for IL-2, 0.99 for IL-6, 0.97 for IFN-γ, and 0.98 for CRP), confirming quantitative robustness in serum.

**Figure 4:**
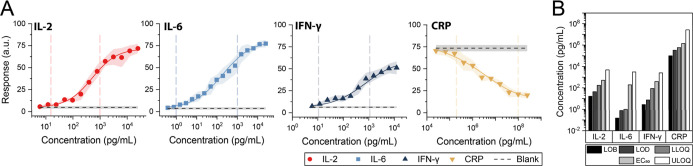
Multiplexed detection of inflammatory biomarkers in human blood serum using the digital nanoplasmonic imaging biosensor. (A) Dose–response calibration curves for IL-2, IL-6, IFN-γ, and CRP. Sigmoidal logistic fits yielded high correlation coefficients (*R* ^2^ = 0.98 for IL-2, 0.99 for IL-6, 0.97 for IFN-γ, and 0.98 for CRP), confirming quantitative performance. For CRP, a monotonic decreasing response was observed, reflecting competition behavior due to high endogenous blood serum CRP levels. Regions between vertical dashed lines highlight clinically relevant detection ranges, demonstrating the capability of PLASMART to cover diagnostic thresholds for both low- and high-abundance biomarkers. (B) Summary of key analytical performance metrics, including limit of blank (LOB), limit of detection (LOD), half-maximal effective concentration (EC_50_), lower limit of quantification (LLOQ), and upper limit of quantification (ULOQ) for each biomarker, confirming low-picogram to low-nanogram sensitivity for cytokines and accurate quantification of CRP across a physiological range of 25 ng/mL to 250 μg/mL .

Unlike the buffer-based experiments described in the above section, CRP in serum exhibited a monotonic decreasing response rather than a biphasic profile. This behavior is attributed to the already elevated endogenous levels of CRP typically present in healthy human serum, ranging from several hundred nanograms per milliliter up to several micrograms per milliliter, which positioned the assay entirely within the competition regime. Consequently, as CRP concentrations increased further, the sensor response progressively decreased due to the saturation and inhibited sandwich formation, consistent with the competition regime described in previous sections.

To further confirm the generality of the biphasic behavior and ultrabroad dynamic range in serum, we performed high-concentration spike experiments with an additional biomarker. As shown in Figure S11, the results with spiked IL-6 in serum displayed a clear bell-shaped response with an intermediate error zone and distinct kinetic signatures for the affinity and competition regimes. These measurements confirm that the competitive dynamics observed in the buffer also persist in serum.

For each biomarker, the shaded regions in Figure 4A highlight the clinically relevant detection ranges, demonstrating that our digital nanoplasmonic biosensor’s dynamic range fully covers, and often exceeds, the diagnostic thresholds necessary for medical applications. Low limits of detection (LOD) in the low picogram-per-milliliter range were achieved for cytokines such as IL-2, IL-6, and IFN-γ, addressing the need for sensitive quantification of low-abundance immune mediators, while the broad dynamic range for CRP addresses both normal and pathological states.

Figure 4B presents the summary of key analytical parameters, including LOB, LOD, EC_50_, LLOQ, and ULOQ for each biomarker. These metrics highlight that the platform maintains robust operation even in serum with subpicogram to low-nanogram per milliliter sensitivity for low-abundance markers while enabling accurate quantification of high-abundance analytes like CRP within physiological ranges (μg/mL range). Additionally, spike–recovery experiments were performed to assess quantitative accuracy in both buffer and human serum. As detailed in Table S2, recoveries ranged from 82.8% to 118.9% across the working range with an average recovery of 103.6%.

### 2.5 Cross-Correlation of the Sensor Performance

We assessed the performance of multiplexed detection with PLASM-ART against clinical multiplexed immunoassay platforms; we used Luminex assay and compared the measurements obtained from five different human blood serum samples (H1–H5), each spiked with four inflammatory biomarkers at clinically relevant concentrations. IL-2, IL-6, and IFN-γ were introduced at increasing concentrations from 6 pg/mL to 25 ng/mL across the five samples, while CRP was spiked at levels ranging from 163 ng/mL to 100 μg/mL. Each measurement was performed in triplicate to check reproducibility and enable for statistical estimation of error.

Figure 5A shows the measured concentrations from both platforms. PLASM-ART measured all four biomarkers in a single run without assay customization. In the Luminex workflow, the middle panel shows results obtained using undiluted serum samples, where cytokines were measurable successfully, but CRP exceeded the upper detection limit. The right panel shows the measurements from Luminex following a 1:500 dilution step, which enabled CRP quantification but lowered the concentration of cytokines below the detection threshold of the assay. These results highlight that simultaneous detection is not possible with Luminex.

**Figure 5:**
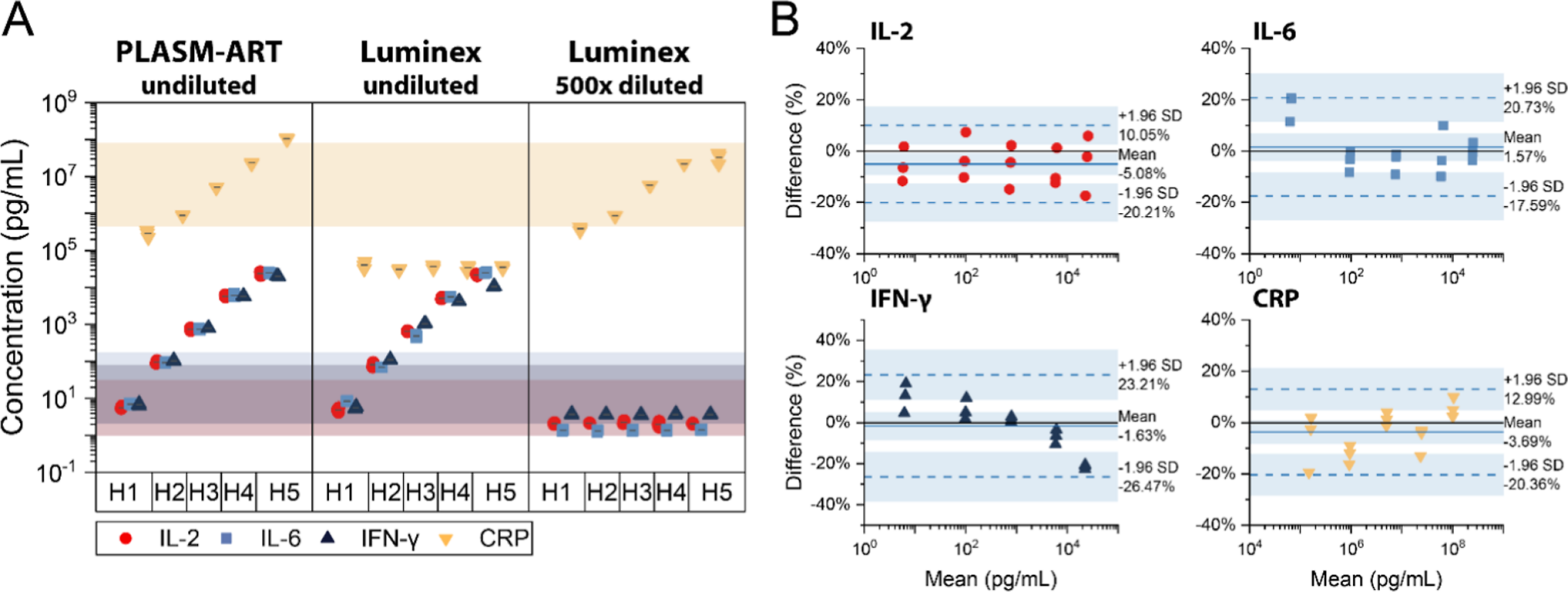
Comparison of the PLASM-ART against a commercial Luminex assay for multiplexed cytokine and CRP detection. (A) Quantification of IL-2, IL-6, IFN-γ, and CRP in human serum samples (H1–H5) measured by the PLASM-ART platform and the Luminex assay under two dilution conditions: undiluted (1:1) and 1:500 dilution. Shaded regions denote the clinically relevant concentration ranges for each biomarker. (B) Bland–Altman analysis comparing PLASM-ART measurements to known spiked concentrations for each analyte. Solid lines indicate the mean bias; dashed lines represent ±1.96 standard deviation (SD) limits. PLASM-ART showed minimal systematic bias of −5.08% (IL-2), 1.57% (IL-6), −1.63% (IFN-γ), and −3.69% (CRP), with limits of agreement ranging approximately ±20% across the full dynamic range, confirming accurate and reproducible quantification.

Interestingly, even with a 1:500 dilution, Luminex exhibited saturation at the highest CRP level (H5 sample, 100 μg/mL), indicating that its effective range remains insufficient to cover the extreme CRP variations observed in clinical settings. This dilution-induced trade-off, between accurately capturing high-abundance markers and retaining sensitivity to low-abundance cytokines, represents a major bottleneck in conventional multiplex assays. In contrast, PLASM-ART captured all biomarker concentrations simultaneously, leveraging its extended dynamic range. The shaded bands in Figure 5A denote clinically relevant ranges for each biomarker, further illustrating that PLASM-ART fully spans the diagnostic window across 9 orders of magnitude without needing parallel workflows or dilution steps, thus facilitating a “one-size-fits-all” biosensing format where routine or extreme CRP levels are measured alongside fg/mL cytokine concentrations.

Individual correlation plots between PLASM-ART and Luminex for each biomarker are shown in the SI (Figure S12A). These plots demonstrate strong linearity for IL-2, IL-6, IFN-γ, and CRP across their respective ranges, confirming the quantitative consistency between the two platforms under suitable operating conditions.

To quantitatively assess the agreement between PLASM-ART measurements and the known spiked concentrations, a Bland–Altman analysis was performed (Figure 5B). The average biases were −5.08% (IL-2), 1.57% (IL-6), −1.63% (IFN-γ), and −3.69% (CRP), with all data points aligned within the ±1.96 SD confidence limits. These results indicate minimal systematic deviation and tight agreement across the entire dynamic range. The minimal variation of differences and no observable concentration-dependent deviation further supports the platform’s accuracy and precision over broad concentration ranges.

In contrast, Bland–Altman plots for Luminex versus spiked concentrations (provided in the SI, Figure S12B) reveal substantially larger biases and broader confidence intervals. The mean differences reached as high as 30.29% for CRP, with wide limits of agreement (+100.92% to −40.34%), reflecting substantial variability and saturation artifacts. Disparities were particularly pronounced at the lowest and highest concentration ranges, where the assay approaches its lower and upper quantification limits. IL-2, IL-6, and IFN-γ showed greater dispersion and higher mean biases compared to PLASM-ART, even at low concentrations, where Luminex typically performs well. Notably, this discrepancy may also arise from the extensive sample dilution required by Luminex assays (1:500), which can introduce nonlinear dilution effects as previously reported.^17^

## 3 Conclusions

In conclusion, in this work, we introduce the PLASM-ART platform, which integrates kinetic profiling of binding events with a digital nanoplasmonic microarray biosensor for enabling multiplexed and quantitative detection of disease biomarkers across an ultrabroad dynamic range spanning in total 9 orders of magnitude, from fg/mL to μg/mL levels, within a simple single-run assay. By digitally registering individual AuNP-labeled binding events on AuNHAs, PLASM-ART captures both low- and high-abundance targets directly from less than 10 μL of unprocessed human serum, without any need for sample splitting or analyte-specific dilution.

Kinetic analysis of the plasmonic images acquired in real-time enables the platform to resolve biphasic dose–response behaviors and to accurately identify transitions between affinity- and competition-dominant regimes, allowing quantification even in the presence of hook-effect interference. This capability ensures reliable measurement across clinically relevant ranges without the need for serial dilutions.

Analytical validation in buffer and serum demonstrated sub-pg/mL sensitivity for cytokines and robust performance for CRP up to 250 μg/mL, with quantification completed within 15 min. Comparison against a clinical Luminex system showed comparable accuracy with minimal systematic bias while eliminating the need for dilution workflows and end point ensemble readouts.

To mitigate cross-reactivity in multiplexed panels, we developed a mechanistic correction framework that analytically decouples three cross-talk pathways. Corrected signals closely matched single-analyte baselines across all targets, confirming a high specificity. The 3D-printed optical reader with an integrated LED and camera for nanoplasmonic detection offers a compact and cost-effective technology. In addition, disposable AuNHA chips are fabricated by using DUVL, enabling high-throughput and scalable production of the sensor substrates.

The synergy of digital detection and kinetic profiling at the core of PLASM-ART unlocks new opportunities for diagnostics in settings where conventional multiplex assays fall short. Its modularity allows for rapid reconfiguration for alternative biomarker panels by adjusting the antibody microarray and nanoparticle labeling scheme. Its digital kinetic detection mode is applicable to a broad range of analytes including cytokines, acute-phase proteins, exosomes, neurodegeneration markers, and cancer antigens. Future development may include integration with automated sample handling, accelerated data acquisition, and machine-learning-assisted kinetic interpretation to support high-throughput clinical workflows.

Furthermore, by uniting single-particle resolution with spatiotemporal kinetic analysis, this work defines a general mechanistic principle governing competitive binding in one-step immunoassays and provides a framework to extend the dynamic range of affinity-based assays across other material, chemical, and biological systems. At the same time, the ability to achieve quantitative multiplex profiling across 9 orders of magnitude in a compact and rapid format highlights the translational potential of this approach, enabling comprehensive biomarker analysis in centralized laboratories as well as decentralized or resource-limited settings. This mechanistic versatility sets the foundation for next-generation immunoassays that are not only more sensitive and informative but also scalable and clinically actionable.

## Supplementary Material



## Notes

Experimental details, materials and methods, nanohole array fabrication and optical setup, sensor surface functionalization and nanoparticle conjugation protocols, buffer and human serum assay procedures, bioassay development and characterization details, calibration models and statistical analysis, kinetic regime classification and competitive surface binding models, cross-reactivity analysis and correction framework, spike-recovery, method comparison studies, and automated image analysis algorithms and workflow (PDF)

## Abbreviations

AuNHA: gold nanohole array
AuNP: gold nanoparticle
cAb: capture antibody
CRP: C-reactive protein
dAb: detection antibody
DUVL: deep ultraviolet lithography
EC_50_: half-maximal effective concentration
EOT: extraordinary optical transmission
ELISA: enzyme-linked immunosorbent assay
H1–H5: human serum samples 1–5
IFN-γ: interferon-gamma
IL-2: interleukin-2
IL-6: interleukin-6
LLOQ: lower limit of quantification
LOB: limit of blank
LOD: limit of detection
PLASM-ART: plasmonic large-dynamic-range analysis sensor for multiplexed assay and rapid tracking
*R* ^2^: coefficient of determination
RSS: residual sum of squares
SD: standard deviation
SEM: scanning electron microscope
ULOQ: upper limit of quantification.
