# Supplementary material for: Kinetic Profiling in One-Step Digital Immunoassays Enables Multiplex Quantification across an Ultrabroad Dynamic Range
Source: J Am Chem Soc. 2026 Jan 27;148(5):5253–63. doi: 10.1021/jacs.5c17838 (PMC12903848; doi:10.1021/jacs.5c17838)
Supplement: Supplementary file 1 [file ja5c17838_si_001.pdf]

Supporting Information (SI) for

Kinetic Profiling in One-step Digital Immunoassays Enables Multiplex Quantification across an Ultrabroad Dynamic Range

Abtin Saateh[1], Rojina Allamehnejad[1], Wenhong Yang[1], Yen-Cheng Liu[1], Genrich V. Tolstonog[2,3], Hatice Altug[1]*

[1]Institute of Bioengineering, École Polytechnique Fédérale de Lausanne (EPFL), CH-1015 Lausanne, Switzerland.

[2]Department of Otolaryngology – Head and Neck Surgery, Lausanne University Hospital and University of Lausanne, Lausanne 1011, Switzerland

[3]AGORA Cancer Research Center, Lausanne 1005, Switzerland

*Email address: hatice.altug@epfl.ch.

Contents

1 Materials and Methods 1
 1.1 Reagents and Materials 1
 1.2 AuNHA fabrication 1
 1.3 Optical Setup 2
 1.4 Sensor Chip Functionalization 2
 1.5 AuNP Functionalization 2
 1.6 Buffer Measurements 3
 1.7 Human Serum Measurements 3
 1.8 Serum Profiling 3
 1.9 Microarray antibody immobilization 4
 1.10 Data acquisition and analysis algorithms 4
 1.11 Statistical methods and LOD calculation 5
2 Bioassay Development 6
 2.1 AuNP Characterizations 6
 2.2 Bound AuNPs on AuNHA 7
 2.3 Binding Buffer Optimization 8
3 Bioassay Characterization 10
 3.1 Logistic Fit Models 10
 3.2 Limit of Blank (LOB) 10
 3.3 Limit of Detection (LOD) 10
 3.4 Limit of Quantitation (LOQ) 11
4 Classification of Binding Regime Based on Association Curves 12
 4.1 Langmuir Binding Model (Reaction-Limited) 12
 4.2 Linear Model (Mass-Transport-Limited) 12
 4.3 Model Fitting and Residual Evaluation 12
5 Competitive Surface Binding Model of NP-Protein Complex and Free Protein 14
 5.1 Diffusion of NP-Protein Complexes vs Excess Protein Analyte 14
 5.2 Effective Association Rate under Mass Transport Limitation 14
 5.3 NP-Protein complex formation in bulk (Equilibrium binding in solution): 15
 5.4 Surface Binding Model: Competitive Langmuir Adsorption 15
6 Cross-reactivity Analysis 19
 6.1 Cross-reactivity Types 19

6.2 Cross-reactivity in Buffer 22
6.3 Cross-reactivity in Human Serum 22
6.4 Cross-reactivity Correction in Human Serum 25
7 Biphasic Behavior of spike IL-6 in Human Serum 26
8 Spike-Recovery Analysis 27
9 Method Comparison 28
9.1 Individual Biomarker Comparison 28
10 Automated Image Analysis 30
10.1 Analysis 30
10.2 Flowchart 32

1 Materials and Methods

1.1 Reagents and Materials

All chemicals were of analytical grade. PBS (pH 7.4), Tween-20, Tris, NaCl, acetone, and isopropanol were purchased from Sigma-Aldrich. Milli-Q water was used for all aqueous preparations (Merck Millipore). MCP coating solution, Block-On blocking buffer, and spotting buffer were obtained from Lucidant. Pierce™ Clear Milk Blocking Buffer (10×) was sourced from Thermo Fisher Scientific. PEGylated 100 nm gold nanoparticles (OD = 20) were purchased from Cytodiagnostics.

Capture and detection antibodies for IL-6 and CRP, as well as corresponding recombinant proteins, were from HyTest. Capture and detection antibodies for IL-2 and IFN-γ, and their recombinant proteins, were from Mabtech. IgG2a isotype control was purchased from Thermo Fisher Scientific. Details of each are summarized in Table S3. An ELISA kit for detecting human anti-mouse antibodies was purchased from ALPCO. All protein samples were handled using Eppendorf Protein LoBind Tubes.

Multiplex cytokine profiling of human serum was performed using the ProcartaPlex Mix & Match 4-plex panel and reagents from Thermo Fisher Scientific, with data acquired on a Luminex™ 200™ system.

1.2 AuNHA fabrication

AuNHAs were fabricated on 4-inch fused silica wafers (500 μm thickness) using a scalable deep ultraviolet lithography (DUVL) and ion beam etching process. Wafers were cleaned using a standard RCA protocol (1:1:5 H_2O_2:NH_4OH:H_2O) for 15 min, then rinsed with deionized water and dried under nitrogen flow. Subsequently, a 10 nm titanium adhesion layer and 120 nm gold film were deposited using an EVA 760 electron-beam evaporator.

A bilayer resist stack comprising a bottom antireflective coating (BARC) and a photoresist (M108Y) was spin-coated and soft-baked. Nanohole arrays (200 nm diameter, 600 nm pitch) were patterned using a 248 nm DUV stepper (ASML PAS 5500/300), followed by resist development. The pattern was first transferred into the resist stack using reactive ion etching (RIE) to open the BARC and expose the underlying gold layer. Subsequently, ion beam etching (IBE; PlasmaLab 300) was used to etch through the gold and titanium layers. Residual resist was removed by oxygen plasma treatment. Wafers were then diced into chips using a precision dicing saw. The

final AuNHA chips were characterized by SEM and optical spectroscopy to confirm plasmonic performance.

1.3 Optical Setup

The nanoplasmonic imager was constructed using the OpenFlexure microscope platform, a modular and open-source design optimized for compact, cost-effective microscopy. All mechanical components, including the stage and optical mounts, were fabricated using a 3D printer. The imaging system employed a 660 nm LED with a 10×/0.3NA objective was used to collect the transmitted light, which had been modulated by the nanohole array sensor. This signal was captured using an IRIS-15 sCMOS camera (Photometrics) at a spatial resolution of ~0.5 μm/pixel. The OpenFlexure microscope frame provided a stable and compact configuration with a footprint of approximately 15 × 15 × 20 cm, supporting precise optical alignment and portability.

1.4 Sensor Chip Functionalization

AuNHAs were cleaned by sequential immersion in acetone, isopropanol, and Milli-Q water (5 min each), dried under nitrogen, and treated with oxygen plasma (Diener Plasma, 1.2 bar, 30 W). MCP stock solutions were freshly diluted 1:50 (v/v) with 5× Coating Solution unless otherwise specified. Chips were immersed in the coating solution for 30 min at room temperature in plastic Petri dishes under gentle shaking. After coating, chips were rinsed with Milli-Q water for 5 min, dried with nitrogen, and cured under high vacuum (<2 mm Hg) at 80°C for 15 min.

Capture antibodies were diluted in spotting buffer to 250 μg/mL and spotted onto MCP-coated AuNHAs within 20 min after baking. Spotting was performed at 22°C and 45% relative humidity, followed by incubation at 55% humidity for 4 hours to ensure efficient coupling and spot morphology. Residual reactive groups were quenched using blocking solution (1:1 Milli-Q water and 2× Block-On) for 30 min at room temperature. Chips were then rinsed with Milli-Q water, dried under nitrogen, and washed in 1× PBS containing 0.1% Tween-20 for 15 min before a final Milli-Q rinse and nitrogen drying.

1.5 AuNP Functionalization

PEG-coated AuNPs (100 nm, OD = 20) were conjugated with detection antibodies of IL-2, IL-6, IFN-γ, and CRP. Antibodies (500 μg/mL) were mixed with reaction buffer and added to lyophilized AuNPs, incubated for 2 h at room temperature with agitation (700 rpm). Quenching was performed

with manufacturer-provided solution, followed by five centrifugation-resuspension cycles (200 rcf, 30 min) in 1× PBS containing 1% BSA and 0.05% Tween20 to ensure the removal of the unreacted antibodies. Final conjugates were stored at 4 °C and vortexed prior to use. Characterizations of AuNPs and AuNP-antibody conjugates were confirmed using TEM, UV-vis and DLS in **Figure S1-S2** and **Table S1**.

1.6 Buffer Measurements

Calibration curves were established using recombinant IL-2, IL-6, IFN-γ, and CRP. Cytokines were tested over a concentration range of 1-25,000 pg/mL, while CRP measurements extended up to 250 µg/mL. Measurement samples were prepared by thawing protein stocks on ice, followed by serial dilution in 1× PBS (pH 7.4). A blank control (1× PBS) was included in each experiment. Final dilutions were vortexed at 800 rpm. All analytes were spiked into the optimized buffer, followed by the addition of the analyte-specific AuNP cocktail.

AuNHA chips were equilibrated to room temperature, fitted with silicone isolators, and blocked with 1× milk-blocking buffer for 3 min. Blocking solution was aspirated carefully, and 10 µL of sample was loaded into each well without allowing the surface to dry. A cover glass was placed and samples were imaged (10× objective, 200 ms exposure). Each concentration was analyzed across at least five microarray spots to calculate mean signal and SD. Binding of AuNP-antibody conjugates to the AuNHA surface was further confirmed by SEM imaging (**Figure S3**).

1.7 Human Serum Measurements

Calibration curves in human serum were established using recombinant IL-2, IL-6, IFN-γ, and CRP. Cytokines were tested over a concentration range of 1-25,000 pg/mL, while CRP measurements extended up to 250 µg/mL.

Serum samples were pre-treated with IgG2a isotype control solution to a final concentration of 1 µg/mL in the assay mixture. All analytes were spiked into the treated serum, followed by the addition of the analyte-specific AuNP cocktail. Each concentration was measured across at least five microarray spots to compute mean signal and SD.

1.8 Serum Profiling

Whole human serum samples were thawed and measured using a 4-plex ProcartaPlex Mix & Match panel (Thermo Fisher Scientific) according to the manufacturer's protocol. Data acquisition

was performed on a Luminex™ 200™ system operated with xPONENT software. Calibration curves were generated using a 5-PL regression model.

1.9 Microarray antibody immobilization

Antibody immobilization was performed using a non-contact piezoelectric ultra-low volume dispenser (cellenONE® X1, Scienion). Spotting conditions were optimized to generate 450-480 pL droplets. During spotting, the chamber humidity was maintained at 30% and the temperature set above the dew point. Immediately before initiating spotting, the humidity was adjusted to 45%. Antibodies specific to IL-2, IL-6, IFN-γ, and CRP were spotted in a 2×2 array format.

1.10 Data acquisition and analysis algorithms

To quantify AuNP signals, we employed an automated image processing pipeline that integrates spot detection, contrast ratio analysis, particle segmentation, and particle-level contrast analysis. A representative example of the processed images, segmented particles, and annotated spots is shown in **Figure S13**.

The complete analysis workflow is outlined in **Figure S14**, which summarizes the modular computational pipeline used for processing images. The pipeline is composed of four interconnected modules: main analysis, CR (contrast ratio) analysis, particle count analysis, and particle contrast analysis. Upon initiation, the system imported necessary libraries, initialized global parameters, and allowed users to select image folders through a graphical interface.

For each acquired image, automatic spot detection was performed using the Circular Hough Transform, followed by manual validation and adjustment steps as needed. Regions of interest (ROIs) were extracted for both spot and background areas, and local mean intensities were calculated. The corrected contrast ratio (CRI) was computed by adjusting for background signals. Annotated images and CRI results were displayed and saved for further analysis.

In parallel, particle count analysis was conducted. Images were preprocessed using adaptive Gaussian thresholding and morphological operations to enhance particle features. A distance transform was applied to aid segmentation, and connected components analysis was used to detect particles. Watershed segmentation further refined particle boundaries. Particle counts and corresponding signal-to-noise ratios (SNR) were computed. Results were displayed, with the possibility of parameter adjustment if detection quality was insufficient.

For particle contrast analysis, each segmented particle was independently evaluated. Pixel intensity data were extracted, and clustering techniques, including K-means clustering, were applied to validate data quality. A contrast ratio for each particle was calculated based on a sigma-adjusted model. If necessary, reprocessing was performed to exclude invalid particles. Particle contrast data were used to generate histograms, compute second moment averages, and produce summary charts and reports.

1.11 Statistical methods and LOD calculation

The sensitivity of the assay was characterized by determining the LOB, LOD, and LOQ, according to established guidelines[1]. The LOB was defined as the highest apparent analyte signal observed when replicates of blank samples containing no analyte were measured. It was calculated as the mean signal of the blank plus 1.645 times its SD (SI Eq. S1). The LOD was defined as the lowest concentration of analyte distinguishable from the LOB (SI Eq. S2). It was calculated by adding 1.645 times the standard deviation of the lowest measured concentration to the LOB signal (SI Eq. S3). To convert the LOD signal into a corresponding analyte concentration, the fitted 5-PL calibration curve was used (SI Eq. S1). The LOD concentration was determined by rearranging the 5-PL equation to solve for concentration at the LOD signal level (SI Eq. S4).

The LOQ was defined as the lowest analyte concentration that could be quantitatively determined with acceptable precision, defined as a coefficient of variation (CV) of less than 5%, and acceptable accuracy. Depending on assay performance, the LOQ may coincide with or be higher than the LOD. The working range of the assay was defined as the interval between the LLOQ and ULOQ where deviations from expected analytical sensitivity were observed.

2 Bioassay Development

2.1 AuNP Characterizations

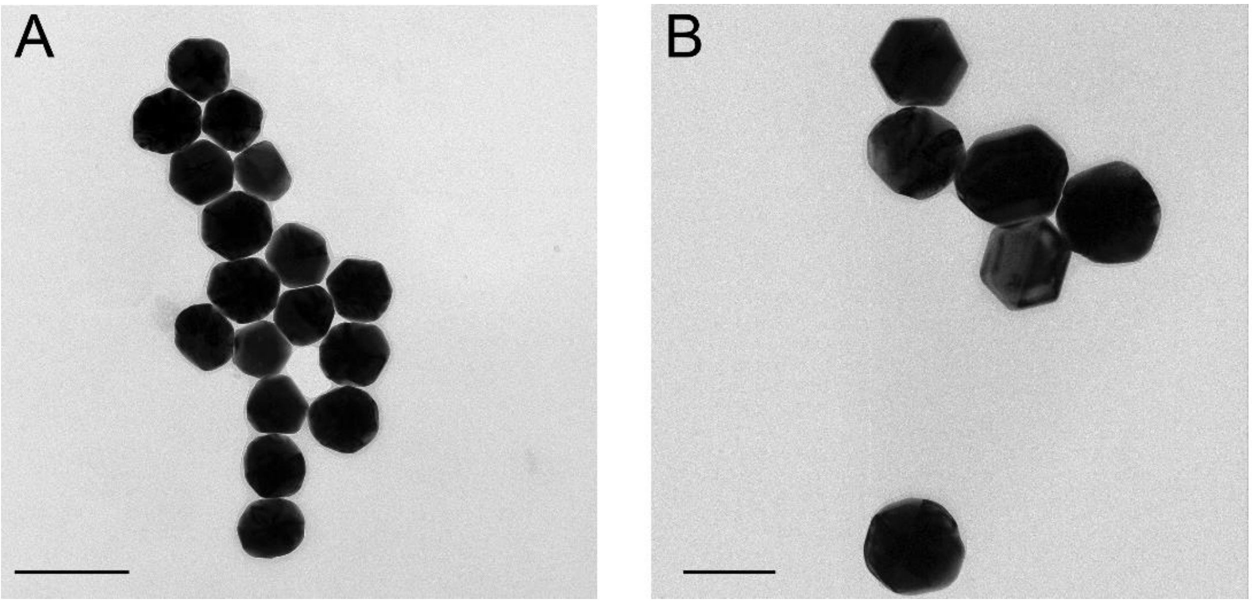

Figure S1. TEM images of the commercial gold nanoparticles (AuNPs) used in this study. (A) Lower-magnification view (200 nm scale bar) and **(B)** higher-magnification view (100 nm scale bar) of the same batch of 100 nm AuNPs.

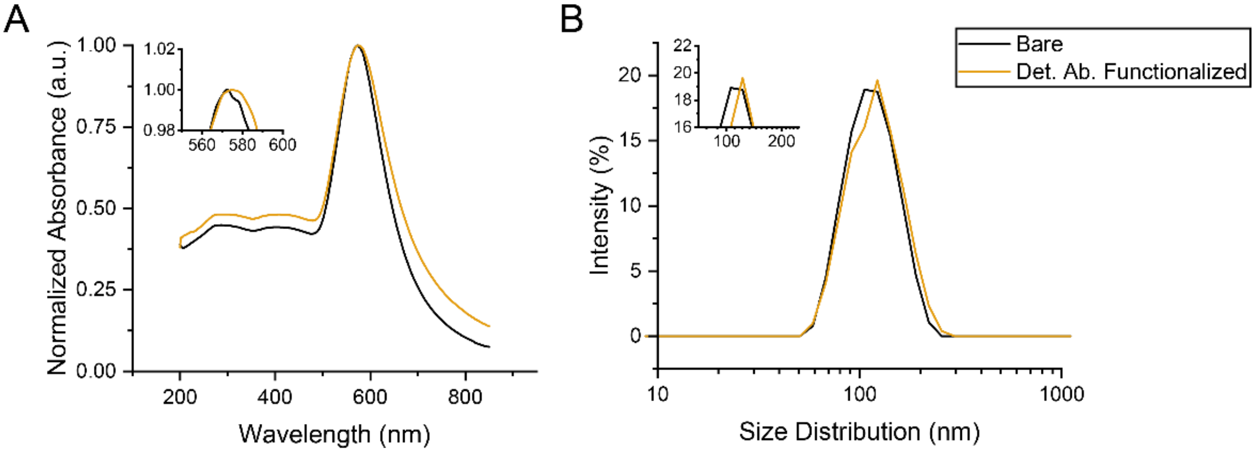

Figure S2. Characterization of 100nm AuNP-antibody conjugates before and after conjugation using **(A)** UV-vis and **(B)** DLS. The insets show the peak region in zoom for better visibility.

Table S1. Summary of UV-vis and DLS, before and after conjugation.

Method	Parameter	Untreated	Det. Ab. Conjugated
UV-vis	LSPR (nm)	571 ± 2	575 ± 2
Zeta Sizer	Hydrodynamic Size (nm)	112 ± 4	120 ± 5
	PDI	4%	3%

2.2 Bound AuNPs on AuNHA

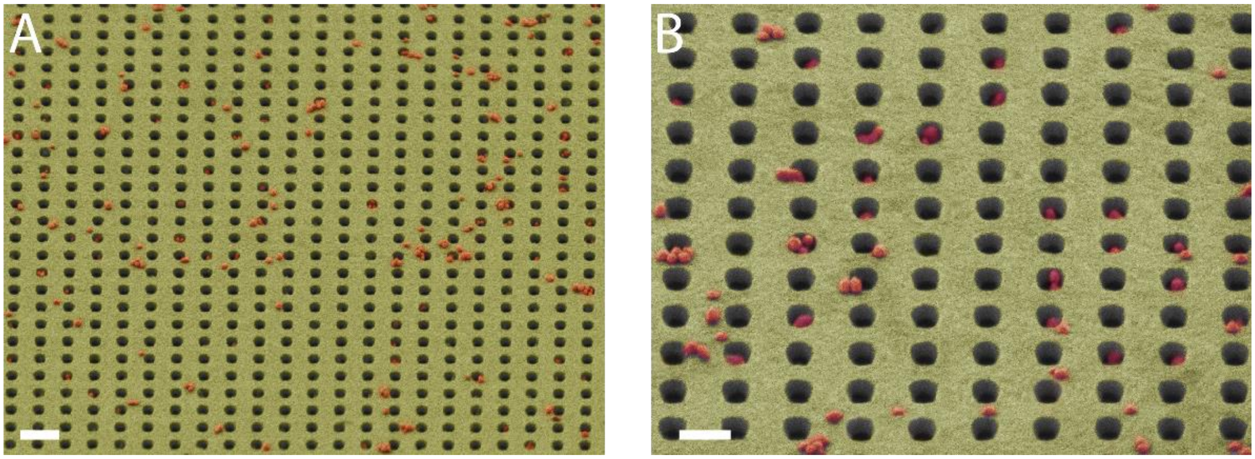

Figure S3. Colored SEM images showing 100 nm AuNPs (red colored) bound to AuNHAs (gold colored) after completion of the sandwich immunoassay. All micrographs were acquired at a 54-degree tilt. The scale bar is 1 µm in the panel **(A)** and 500 nm in the panel **(B).**

2.3 Binding Buffer Optimization

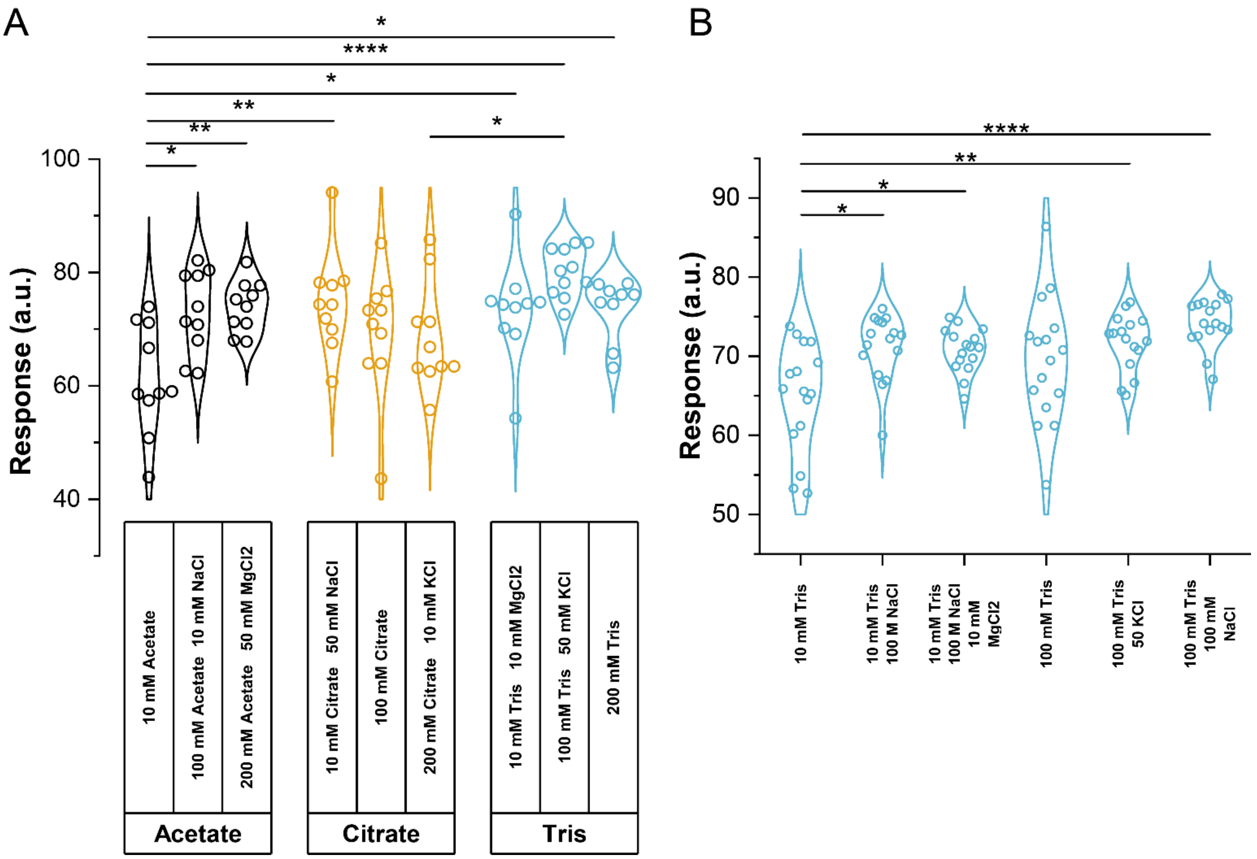

Figure S4. Comparison of binding buffer screening experiments to maximize specific signal and minimize variability. Violin plots represent the distribution of sensor responses for IL-6 at a concentration of 12.5 ng/mL across various buffer compositions. Each point represents an individual replicate measurement. The width of each violin reflects the probability density of the data at different response values. **(A)** A Taguchi designed buffer compositions across three different buffer types: Acetate, Citrate, and Tris, with varying salt additives (NaCl, $MgCl_2$, KCl). **(B)** Fine-tuning of the Tris buffer by further optimizing salt concentration and type.

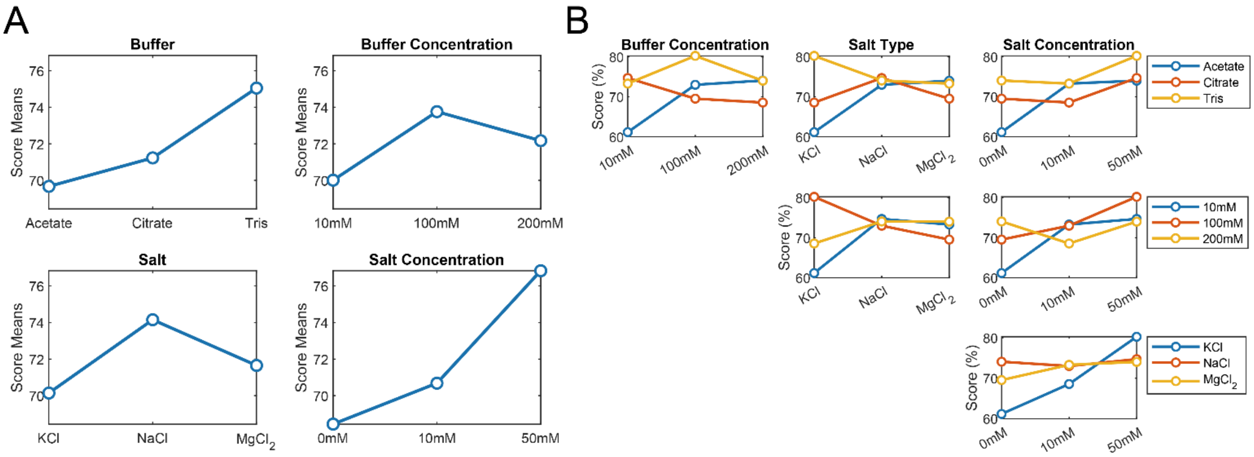

Figure S5. Taguchi-based analysis of binding buffer optimization. (A) Main effects plot showing the influence of four parameters, buffer type, buffer concentration, salt type, and salt concentration, on the mean sensor response. Tris buffer, 100 mM concentration, NaCl as the salt, and 50 mM salt concentration yielded the highest average responses, indicating favorable conditions for IL-6 binding. **(B)** Interaction plots depict how combinations of parameters interact to affect the assay signal. Notably, buffer type and salt concentration exhibited a strong interaction, with Tris buffer and 50 mM salt concentration resulting in synergistically higher responses.

3 Bioassay Characterization

This section describes the mathematical models and analytical criteria used for data fitting and for calculating the key performance metrics: Limit of Blank (LOB), Limit of Detection (LOD), and Limit of Quantitation (LOQ) are calculated based on CLSI EP17-A2 guideline[1].

3.1 Logistic Fit Models

To model dose-response calibration curves, the 4-PL and 5-PL model are used:

$$\text{4-PL: } y = R_{min} + \frac{R_{max} - R_{min}}{1 + \left(\frac{C}{K_D}\right)^n} \quad \text{Eq. S1}$$

$$\text{5-PL: } y = R_{min} + \frac{R_{max} - R_{min}}{\left(1 + \left(\frac{C}{K_D}\right)^n\right)^s}$$

where:

R_{min} is minimum response, R_{max} is maximum response, K_D is target analyte concentration at inflection point (EC_{50}), n is slope of the fit (Hill slope), and s is asymmetry parameter.

3.2 Limit of Blank (LOB)

LOB is the highest apparent analyte concentration expected to be found when replicates of a blank sample containing no analyte are tested.

$$LOB_{signal} = \overline{Signal_{blank}} + 1.645 \times SD_{blank} \quad \text{Eq. S2}$$

where:

$\overline{Signal_{blank}}$ is mean signal of blank replicates and SD_{blank} is standard deviation of blank signals.

3.3 Limit of Detection (LOD)

The LOD is the lowest analyte concentration that yields a signal distinguishable from the blank with 95% confidence. First, the LOD signal is estimated:

$$y = LOD_{signal} = LOB_{signal} + 1.645 \times SD_{low} \quad \text{Eq. S3}$$

where:

SD_{low} is the standard deviation of the lowest non-zero concentration tested.

The corresponding LOD concentration is obtained by inverting the logistic fit model (Eq. S1). Solving for C (concentration) at $y = LOD_{signal}$ gives $LOD_{concentration}$.

3.4 Limit of Quantitation (LOQ)

LOQ is the lowest concentration of analyte in a sample which can be quantitatively determined with suitable precision (CV < 5%) and accuracy. The LOQ may be equivalent to the LOD or it could be at a much higher concentration.

4 Classification of Binding Regime Based on Association Curves

To determine the dominant kinetic regime, whether reaction-limited or mass-transport-limited association kinetics are observed, we implemented a model fitting and residual analysis pipeline. Each dataset was fit to three kinetic models representing distinct binding mechanisms:

4.1 Langmuir Binding Model (Reaction-Limited)

Assumes analyte binding is limited by molecular interaction kinetics. The response $R(t)$ over time follows:

$$R(t) = R_{max}\left(1 - e^{-(k_{on}C + k_{off})t}\right) \quad \text{Eq. S4}$$

where:

- R_{max} is the maximum binding response,
- k_{on} and k_{off} are the association and dissociation rate constants, respectively,
- C is the analyte concentration.

4.2 Linear Model (Mass-Transport-Limited)

Assumes binding is limited by the rate at which analyte reaches the surface. The signal rises according to:

$$R(t) = R_{max}\left(1 - e^{-k_t t}\right) \quad \text{Eq. S5}$$

Where k_t is the apparent mass transport rate constant.

As mass transport becomes severely limiting, binding rate is capped by diffusive flux, which is a linear growth in time.

Used as a benchmark for transport-limited behavior, especially in short-time regimes where the response appears approximately linear:

$$R(t) = D\frac{C_b}{\delta}t \quad \text{Eq. S6}$$

where D is the diffusion constant, C_b is the analyte concentration in bulk, and δ is diffusion boundary layer thickness.

4.3 Model Fitting and Residual Evaluation

Each model was independently fit to the baseline-corrected association phase of each dataset using non-linear least squares. The residual sum of squares (RSS) was calculated for each fit as:

$$RSS = \sum_{i=1}^{n} \left[R_{obs}(t_i) - R_{fit}(t_i)\right]^2 \qquad \text{Eq. S7}$$

where $R_{obs}(t_i)$ is the observed response at time t_i, and $R_{fit}(t_i)$ is the model-predicted response.

Regime Classification Criteria

The binding regime for each dataset was assigned based solely on RSS comparisons between the Langmuir and linear models, following the logic:

- If $RSS_{Langmuir} < 0.5 \times RSS_{Linear}$ then we have Reaction-limited regime
- If $RSS_{Linear} < 0.5 \times RSS_{Langmuir}$ then we have Mass-transport-limited regime
- Otherwise, we have Mixed regime.

This threshold ensures that only fits with a clear superiority in describing the observed data are assigned to a dominant kinetic regime. The mass-transport-limited exponential model was also fit to provide a physically meaningful comparison curve, but not used directly in the classification rule.

5 Competitive Surface Binding Model of NP-Protein Complex and Free Protein

5.1 Diffusion of NP-Protein Complexes vs Excess Protein Analyte

The diffusion coefficients of analytes were estimated using the Einstein-Stokes relation:

$$D = \frac{k_B T}{6\pi\eta r} \quad \text{Eq. S8}$$

where:

- k_B is the Boltzmann constant (1.38×10^{-23} J/K),
- T is the absolute temperature (assume room temperature, ~298 K),
- η is the dynamic viscosity of the fluid (for water at room temperature, $\sim10^{-3}$ Pa·s),
- r is the radius of the spherical particle.

Estimated diffusion coefficients:

- 5 nm (corresponding to a representative molecule): $D \approx 8.73 \times 10^{-11}\ {m^2}/{s}$
- 100 nm (corresponding to the used AuNP size in the experiments): $D \approx 4.36 \times 10^{-12}\ {m^2}/{s}$

This ~20-fold difference in diffusion rates indicates that free proteins reach the surface significantly faster than nanoparticle-protein complexes under diffusion-based conditions.

5.2 Effective Association Rate under Mass Transport Limitation

The effective association rate constant ${k_{on}}^{\text{eff}}$ accounts for both reaction kinetics and diffusion transport limitations[2,3] and is defined as:

$${k_{on}}^{\text{eff}} = \left(\frac{1}{{k_{on}}^{intrinsic}} + \frac{1}{{k_{on}}^{mt}}\right)^{-1} \quad \text{Eq. S9}$$

where:

- ${k_{on}}^{intrinsic}$ is the reaction-limited association rate constant [$M^{-1}s^{-1}$],
- ${k_{on}}^{mt}$ is the diffusion-based association rate constant [$M^{-1}s^{-1}$].

Mass transport to the surface is modeled using a boundary-layer approximation. The mass-transfer coefficient k_t is:

$$k_t = \frac{D}{\delta} \qquad \text{Eq. S10}$$

where, δ is the diffusion boundary-layer thickness (here assumed as 100 µm[4–6]).

The mass-transport-limited pseudo second-order rate constant is:

$$k_{on}{}^{mt} = \frac{k_t}{\Gamma_{max}} \times 10^3 \qquad \text{Eq. S11}$$

where, Γ_{max} is the maximal surface density (mol/m^2), and factor 10^3 converts to units of $M^{-1}s^{-1}$. Here we assumed 0.1 pmol/cm^2.[7]

5.3 NP-Protein complex formation in bulk (Equilibrium binding in solution):

Before surface binding, NP-protein complexes form in solution according to a Langmuir equilibrium:

$$C_1 = C_{1,NP} N_b \frac{C_2}{C_2 + K_D^{NP}} \qquad \text{Eq. S12}$$

where:

- C_1 is the concentration of NP-protein complexes [M],
- $C_{1,NP}$ is the total NP concentration [M],
- N_b is number of IgG molecules per NP (computed from NP surface area and IgG footprint)
- C_2 is the concentration of free protein [M],
- K_D^{NP} is the dissociation constant of protein-NP binding [M].

This step assumes rapid equilibrium compared with surface adsorption, consistent with typical pre-binding or labeling behavior.

5.4 Surface Binding Model: Competitive Langmuir Adsorption

Surface binding is modeled as a two-species competitive Langmuir adsorption process, where both species (NP-protein complex and free protein) compete for the same capture antibody sites:

$$\frac{d\theta_1}{dt} = k_{on,1}{}^{\text{eff}} C_1(1 - \theta_1 - \theta_2) - k_{off,1}\theta_1$$

$$\frac{d\theta_2}{dt} = k_{on,2}{}^{\text{eff}} C_2(1 - \theta_1 - \theta_2) - k_{off,2}\theta_2$$

Eq. S13

where:

- θ_1, θ_2 are surface coverage by NP-protein complexes and free proteins, respectively (dimensionless, range: 0-1),
- $k_{on,1}{}^{\text{eff}}$, $k_{on,2}{}^{\text{eff}}$ are diffusion-limited association rate constants [$M^{-1}s^{-1}$],
- $k_{off,1}$, $k_{off,2}$ are dissociation rate constants [s^{-1}],
- C_1, C_2 are bulk concentrations of NP-protein complexes and free proteins [M],
- C_2 is the fraction of available surface binding sites.
- Available surface sites are shared: $\theta_1 + \theta_2 \leq 1$

with initial conditions: $\theta_1(0) = \theta_2(0) = 0$

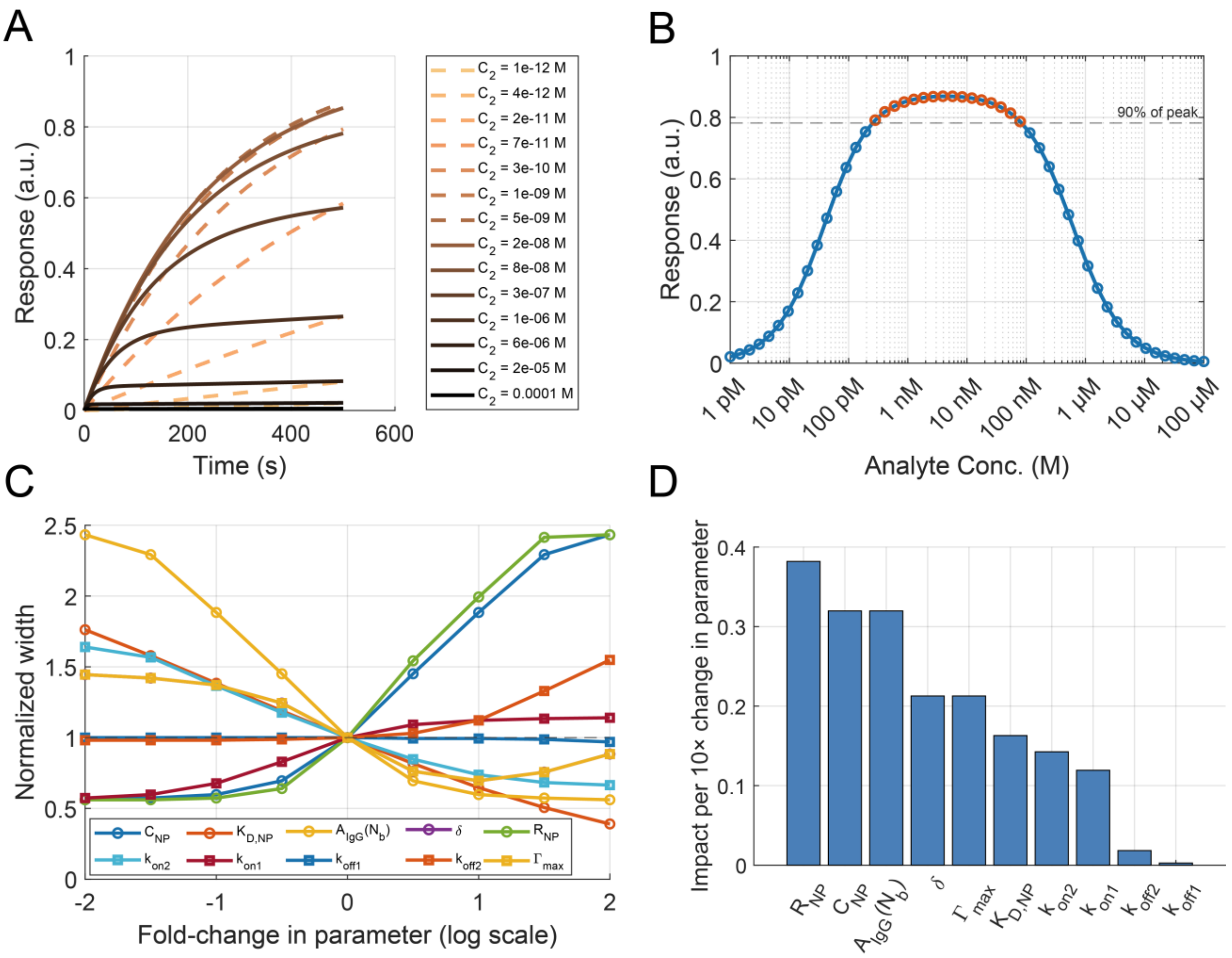

Figure S6. Analytical nanoparticle- excess protein competition on a capture surface under combined kinetic and mass-transport limitations. Simulations were performed using base parameters representative of nanoparticle-enhanced biosensing: nanoparticle concentration $C_{1,NP} = 10^{-9}$ M, NP-protein dissociation constant $K_D^{NP} = 10^{-10}$ M, IgG footprint $A_{\text{IgG}} = 30\ \text{nm}^2$, nanoparticle radius $R_{NP} = 50$ nm, protein radius $R_{NP} = 2.5$ nm, mass-transport boundary-layer thickness $\delta = 10^{-4}$ m, intrinsic association rates $k_{on,1\ (\text{NP-Ag})}^{intrinsic} = k_{on,2\ (\text{protrein})}^{intrinsic} = 10^4\ \text{M}^{-1}\text{s}^{-1}$, dissociation rates $k_{\text{off},1\ (\text{NP-Ag})}^{intrinsic} = 5 \times 10^{-6}\ \text{s}^{-1}$, $k_{\text{off},2\ (\text{protein})}^{intrinsic} = 5 \times 10^{-4}\ \text{s}^{-1}$, and surface site density $\Gamma_{max} = 10^{-9}\ \text{mol/m}^2$. **(A)** Kinetic response of the time dependent binding events for 14 concentrations of target protein in the range of ($C_2 = 10^{-12}$ - 10^{-4} M). Dashed curves denote concentrations for increasing NP binding and solid curves are for NPs competing with excess proteins. **(B)** Concentration-response curve $\theta_1(t_{end})$ obtained at t = 500 s for 50 concentrations, assuming the error-zone as the fraction of concentrations producing ≥ 90% of the maximum signal. **(C)** Sensitivity analysis of the error-zone width. Each curve shows how the normalized width changes when a given physical parameter is varied uniformly from 0.01× to 100× its base value (as specified in the first part of this caption). For each parameter value, the bell-shaped response curve is evaluated and its width in concentration decades (defined as the $\log_{10}$-span of analyte concentrations producing ≥90% of the maximal NP signal) is computed and normalized with its base value. A value of 1 on the y-axis indicates no change from the baseline; values >1 indicate a broader bell region, whereas values <1

indicate a narrower bell. In this panel, identifying the *shape* and *steepness* of each curve provide insights on the influence of a given parameter. For example, parameters whose curves rise sharply with fold-change (e.g., R_{NP}, IgG footprint A_{IgG}, and nanoparticle concentration $C_{1,NP}$) exert strong control over the width, while flatter curves (e.g., k_{on1}, k_{on2}, k_{off1}, k_{off2}) indicate minimal influence. **(D)** Ranked local sensitivities, computed as the magnitude of change in log(width)/ change in log(parameter) near the baseline, which quantifies how much the bell width responds to a tenfold change in each parameter. A sensitivity value of S means that increasing parameter p by a factor of 10 changes the bell width by approximately 10^{S}-fold. Larger bars denote parameters with strongest control over the bell-shaped region width. The analysis indicates that NP size (R_{NP}), IgG footprint, and $C_{1,NP}$ emerge as dominant contributors, whereas intrinsic binding kinetics contribute comparatively weakly.

6 Cross-reactivity Analysis

6.1 Cross-reactivity Types

To characterize and correct for cross-reactivity in the multiplexed biosensor, we performed a detailed analysis using both single-analyte and multi-analyte tests. A conceptual overview of the five key cross-reactivity mechanisms considered in this study is shown schematically in **Figure S7.** In the ideal binding pathway, a detection antibody (dAb) recognizes its cognate analyte, and the resulting immune complex is captured exclusively by the matching capture antibody (cAb). The remaining five pathways represent distinct sources of analytical error. In a dAb-analyte event the dAb binds a non-target analyte which is then sequestered by its own cAb, elevating the signal on the wrong spot. A dAb-cAb event is the converse: the dAb adsorbs directly to an off-target cAb in the absence of analyte. In a cAb-analyte event a free analyte binds an off-target cAb and recruits its cognate dAb. The final two pathways, dAb-dAb aggregation and analyte-analyte bridging, can, in principle, generate signal through higher-order clustering but were not detected under any condition tested. Neither visible precipitates nor concentration-dependent baseline drifts were observed, and no heteromeric complexes of IL-2, IL-6, IFN-γ or CRP have been reported at the concentrations used. Both pathways were therefore excluded from further analysis.

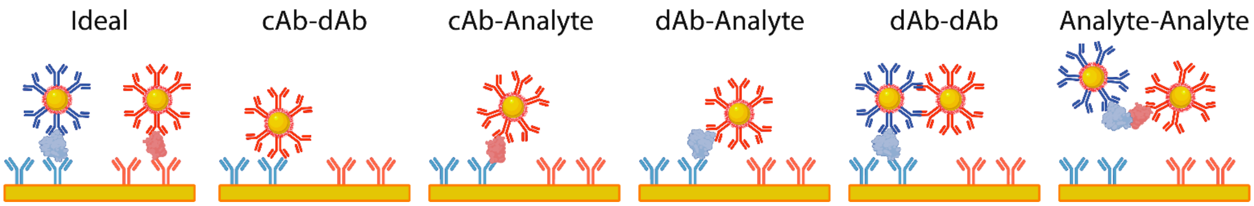

Figure S7. Schematic representation of reagent-based cross-reactivity types in multiplexed immunoassays. Six potential interaction modes are illustrated that contribute to cross-reactivity in sandwich-type assays using detection antibodies (dAbs). Ideal scenario: The detection antibody (blue) and analyte (blue) bind specifically to the corresponding capture antibody (blue) immobilized on the surface. cAb-dAb: The detection antibody interacts directly with an off-target capture antibody (blue), in the absence of any analyte. cAb-Analyte: An off-target analyte binds nonspecifically to a non-cognate capture antibody. dAb-Analyte: The detection antibody binds nonspecifically to an off-target analyte (red), resulting in signal generation despite absence of its cognate target. dAb-dAb: Detection antibodies aggregate via nonspecific interactions, forming sandwich-like structures without target involvement. Analyte-Analyte: Analyte dimers or complexes bridge unrelated capture and detection antibodies, producing spurious signal. Each non-ideal mode represents a distinct mechanism of cross-reactivity that can lead to signal artifacts in multiplexed systems.

To formalize the contribution of the three operative mechanisms we consider the observed multiplex signal on spot i (S_{ii}^{multi}) as the sum of the single-analyte response (S_{ii}^{single}) and the weighted cross-reactive contributions of every non-cognate analyte j:

$$S_{ii}^{multi} = S_{ii}^{single} + CR_{ij}^{cAb-dAb}.S_{ii}^{single} + \sum_{j\neq i}\left(CR_{ij}^{cAb-analyte} + CR_{ij}^{dAb-analyte}\right).S_{jj}^{single} \qquad \text{Eq. S15}$$

where:

- S_{ii}^{multi} is the observed signal in a multiplex format on spot i
- S_{ii}^{single} is the true (corrected) signal from analyte i (its own cognate spot)
- S_{jj}^{single} is true signal of analyte j measured on capture spot j (its own cognate spot)
- $CR_{ij}^{cAb-dAb}$, $CR_{ij}^{cAb-analyte}$, and $CR_{ij}^{dAb-analyte}$ are the respective cross-reactivity coefficients

The three dimensionless cross-reactivity coefficients are defined as follows. For detection antibody-capture antibody (dAb-cAb) interactions, we measured the baseline signal on each capture spot in the absence of analyte (blank condition) using the full mixture of dAb-AuNP conjugates. To express this as a dimensionless cross-reactivity coefficient, the baseline signal was normalized to the single-analyte signal on the same spot at the EC_{50} concentration:

$$CR_{i}^{cAb-dAb} = \left(\frac{S_{ii}^{blank}}{S_{ii}^{single}\Big|_{EC50}}\right) \qquad \text{Eq. S16}$$

This coefficient reflects the cumulative nonspecific adsorption of all dAb-AuNPs to capture spot i, even in the absence of target analyte. A positive value indicates weak but measurable off-target binding, such as Fc-Fc interactions, that can elevate background signal. A value near zero suggests negligible nonspecific binding from the detection cocktail.

For cAb-Analyte interactions, we assessed off-target recognition by titrating each analyte individually in the presence of the full panel of dAb-AuNP conjugates. Each analyte i was titrated individually, and the resulting signals on off-target capture spots j were normalized to the signal on the cognate spot i under identical conditions:

$$CR_{ij}^{cAb-analyte} = \left(\frac{S_{ij}^{single}}{S_{ii}^{single}} \right) \qquad \text{Eq. S17}$$

where S_{ij} is the signal on spot j in response to analyte i, and S_{ii} is the signal from analyte i on its cognate spot under identical conditions. Because all detection antibodies are present during each assay, signal formation requires that analyte i bind directly to capture antibody j in order to be recognized by its matching dAb. Thus, a nonzero value of $CR_{ij}^{cAb-analyte}$ reflects the ability of analyte i to bind non-cognate capture antibody j, enabling sandwich complex formation and producing a false-positive signal. Detection antibody-analyte (dAb-Analyte) interactions alone cannot generate signal on an off-target capture spot unless analyte i is first immobilized by cAb j; this formulation therefore isolates the effect of cAb-Analyte cross-reactivity.

If detection antibody j binds non-cognate analyte i, the resulting complex can be captured on spot i, leading to an increased signal at spot i under multiplexed conditions. The dAb-analyte coefficient cannot be isolated directly; it is extracted from the multiplex data by deducting the two preceding contributions.

The resulting signal elevation on spot *i* reflects a detection-level cross-reactivity and is quantified as:

$$CR_{ij}^{dAb-analyte} = \frac{S_{ii}^{multi} - \left(1 + CR_{i}^{cAb-dAb}\right) S_{ii}^{single} - \sum_{j\neq i} CR_{ij}^{cAb-analyte} . S_{jj}^{single}}{\sum_{k\neq i} S_{kk}^{single}} \qquad \text{Eq. S18}$$

Because several interferents act concurrently, their individual influence is apportioned according to their relative single-analyte intensities, $\omega_j = \frac{S_{jj}^{single}}{\sum_{k\neq i} S_{kk}^{single}}$. Substituting ω_j yields

$$CR_{ij}^{dAb-analyte} = \frac{S_{jj}^{single}}{\sum_{k\neq i} S_{kk}^{single}} . \left(\frac{S_{ii}^{multi} - S_{ii}^{single} - \sum_{j\neq i}\left(CR_{ij}^{dAb-cAb} + CR_{ij}^{cAb-analyte}\right) . S_{jj}^{single}}{\sum_{k\neq i} S_{kk}^{single}} \right) \qquad \text{Eq. S19}$$

Positive values indicate the formation of such complexes and corresponding false-positive contributions. In contrast, negative values arise when non-cognate analytes act as competitive inhibitors-binding to dAb j but failing to produce a measurable signal, effectively suppressing the correct response. Such signal suppression may occur in the presence of excess interferents and

reflects a competitive binding dynamic. A zero value suggests that dAb i does not bind to any non-cognate analytes under multiplex conditions.

6.2 Cross-reactivity in Buffer

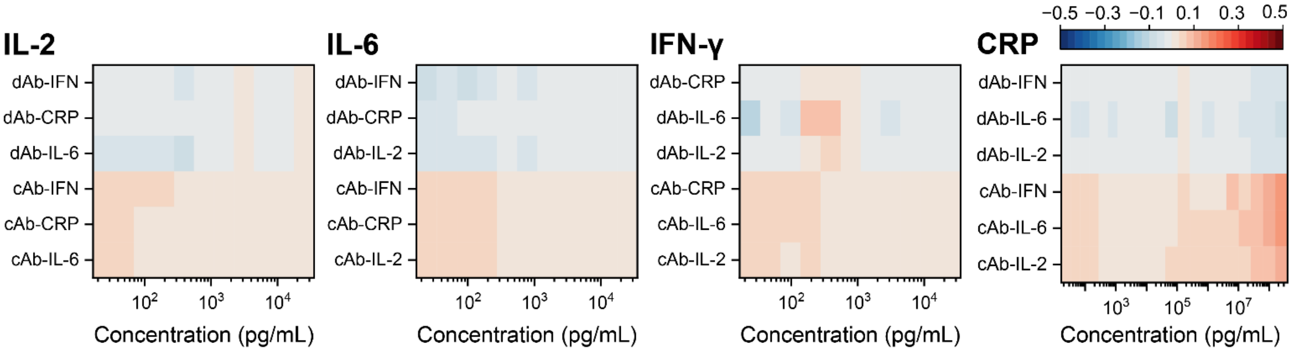

Figure S8. Extracted cross-reactivity coefficients in buffer for each biomarker channel, shown as heatmaps across a wide concentration range. Coefficients correspond to analyte binding to off-target capture antibodies (cAb-analyte) and detection antibody binding to non-cognate analytes (dAb-analyte).

6.3 Cross-reactivity in Human Serum

While an ideal sandwich immunoassay achieves high specificity through selective capture-detection antibody interactions, human serum introduces a complex array of interferants that can bridge, block, or nonspecifically bind assay components (**Figure S9A**). Transitioning from buffer to serum, our sensor maintained excellent specificity. While background signals were slightly elevated in serum, antifouling surface chemistries and careful antibody optimization effectively suppressed nonspecific interactions to levels that did not interfere with analyte quantification. A notable background signal was observed at the IL-2 capture spot in some healthy donor samples (**Figure S9B**), despite the absence of detectable IL-2 as confirmed by Luminex assays (LOD <3 pg/mL). In contrast, CRP capture spots yielded strong signals consistent with physiological CRP levels. Control experiments using fetal bovine serum (FBS) revealed negligible IL-2 background, indicating that the interference was specific to human serum components (**Figure S9B**). Additional spiking experiments confirmed that recombinant IL-2 induced concentration-dependent responses in FBS and buffer but not in human serum, suggesting an alternative source of signal, plausibly due to heterophilic antibodies can disrupt assay. Indeed, heterophilic antibodies are a major concern in immunoassay development, with multiple technical notes and reports highlighting their negative impact[8–15]. **Figure S9C** elaborates mechanistically on how bridging interference enables direct cross-linking between capture and detection antibodies, inflating

signal even in the absence of analyte. Conversely, inhibition interference occurs when interfering antibodies sterically hinder detection antibody access to the target epitope, suppressing true signal. To quantify the extent of such interference, we employed an ELISA-based HAMA detection assay (**Figure S9D**). Although HAMA levels in tested donors were typically low, they occasionally reached up to ~70 ng/mL. The reported prevalence of heterophilic antibodies in healthy individuals varies markedly, ranging from ~1-2% to as high as 40% in asymptomatic individuals[16,17], typically in the range of tens of ng/mL to hundreds of ng/mL depending on the sensitivity of the detection method[18]. These levels, while subclinical, are sufficient to cause signal distortion in sensitive detection formats, highlighting the need for proactive mitigation strategies.

To suppress HAMA-induced cross-reactivity, we implemented a competitive blocking strategy using murine isotypes. We evaluated both mouse IgG1 and IgG2a subclasses and found that IgG2a more effectively mitigated nonspecific signals at equivalent concentrations. This observation aligns with findings by Dekkers et al., who reported markedly higher binding affinities of human IgG to murine IgG2a compared to IgG1, likely due to subclass-dependent interactions with Fcγ receptors[19]. Titration experiments (**Figure S9E**) revealed that 1 µg/mL IgG2a effectively reduced nonspecific IL-2 signals without compromising CRP detection. Higher concentrations (≥2 µg/mL) further suppressed background but also partially reduced specific CRP responses, establishing 1 µg/mL as the optimal blocking concentration neutralizing endogenous interferents and preserving assay performance. Similar observations were made by others, who reported successful removal of false-positive signals induced by heterophilic antibodies from patient plasma by preincubating samples with animal-derived IgG in a sandwich ELISA system[20].

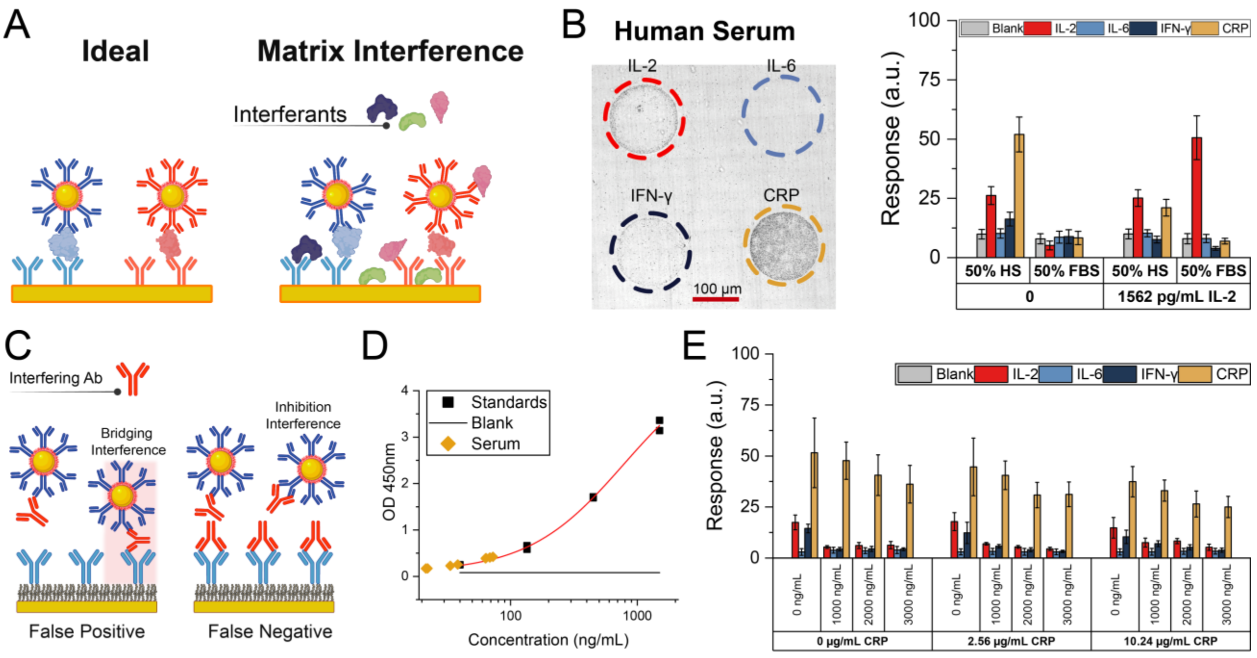

Figure S9. Matrix-based interference and mitigation strategies in multiplexed immunoassays. (A) Schematic of an ideal sandwich immunoassay (left) versus matrix interference (right), where endogenous serum components disrupt antibody-analyte binding, causing off-target signals. **(B)** Left: Sensor image in 50% human serum (HS) without spiked analytes shows strong IL-2 signal and endogenous CRP. Right: Signal comparison in 50% HS vs. fetal bovine serum (FBS) with and without IL-2 spike (1562 pg/mL). In HS, IL-2 signal is elevated even without spiking, and spiking reduces CRP signal, indicating interference. FBS shows clean IL-2 response and low background. **(C)** Mechanisms of heterophilic antibody interference: bridging (false positives) and inhibition (false negatives). **(D)** ELISA-based HAMA calibration (OD at 450 nm) shows a sigmoidal response from 0-1500 ng/mL. Most serum samples had low HAMA, though some reached ~70 ng/mL. Standards and samples were measured in duplicate. **(E)** IgG2a titration blocks nonspecific IL-2 signal; 1 µg/mL IgG2a effectively quenches background without affecting CRP detection. Error bars show standard deviations from triplicates.

6.4 Cross-reactivity Correction in Human Serum

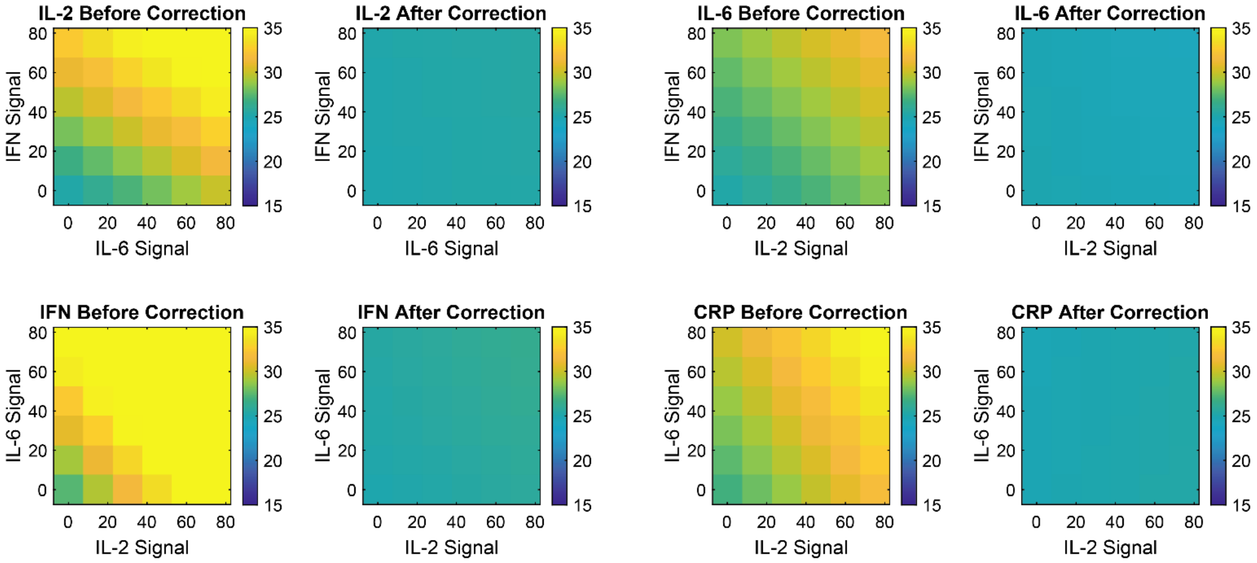

Figure S10. Correction framework across all biomarkers. Heatmaps show raw and corrected signal responses for IL-2, IL-6, IFN-γ, and CRP under multiplexed conditions as a function of co-analyte signal levels. Before correction (left panels), IL-2, IL-6, IFN-γ, and CRP signals were variably affected by off-target interference. After applying the correction algorithm (right panels), all biomarker responses became independent of non-cognate analyte concentrations, yielding flat response surfaces consistent with single-analyte behavior.

7 Biphasic Behavior of spike IL-6 in Human Serum

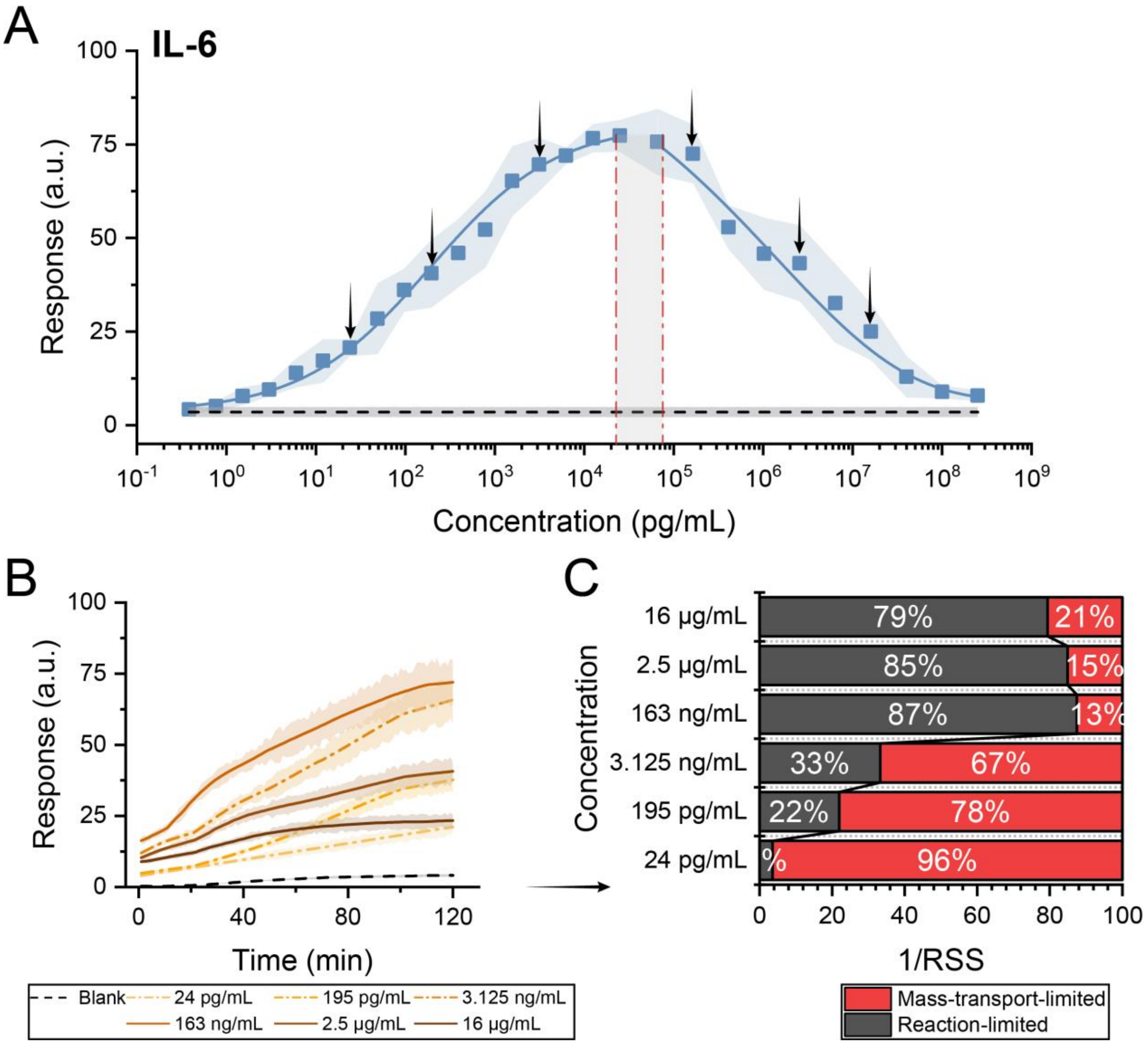

Figure S11. Biphasic binding behavior and kinetic regime analysis for IL-6. (A) IL-6 exhibited a biphasic dose-response profile, with an intermediate “error zone” separating affinity and competition regimes; the boundaries of this zone are marked with dot-dash vertical lines, corresponding respectively to the ULOQ of the affinity regime and the LLOQ of the competition regime, and each regime was fitted independently. **(B)** Kinetic response of the time dependent binding events for six representative IL-6 concentrations (indicated with black arrows in panel A) reveal distinct association profiles characteristic of the affinity (low concentration) and competition (high concentration) regimes. **(C)** Binding regime analysis using the inverse relative normalized residuals (1/RSS), enabling discrimination between underlying binding mechanisms even when endpoint signals converge.

8 Spike-Recovery Analysis

We prepared spiked samples at selected concentrations, performed the assay to measure their responses, and calculated recovery as the ratio of measured to spiked concentration (×100%), using triplicate measurements.

Table S2. Spike recovery test results at several selected concentrations for each biomarker both in buffer and human serum.

Matrix	Target	Conc. (pg/mL)	Recovery (%)	Average Recovery of each biomarker
Buffer	IL-2	5E+1	110.7 ± 5.5	111.2 ± 4.9 %
		5E+2	104.1 ± 1.9	
		5E+3	118.9 ± 7.2	
	IL-6	5E+1	97.3 ± 12.0	104.6 ± 7.1 %
		5E+2	102.5 ± 2.8	
		5E+3	113.9 ± 6.4	
	IFN-γ	5E+1	116.7 ± 6.0	112.1 ± 4.2 %
		5E+2	101.5 ± 2.8	
		5E+3	118.3 ± 3.9	
	CRP	5E+1	100.7 ± 8.3	101.3 ± 6.2 %
		5E+2	86.7 ± 2.4	
		5E+3	86.0 ± 4.2	
		1E+6	112.6 ± 6.5	
		1E+7	117.7 ± 5.7	
		1E+8	104.3 ± 10.2	
Serum	IL-2	5E+1	118.0 ± 10.2	103.0 ± 5.6 %
		5E+2	99.3 ± 1.9	
		5E+3	91.7 ± 4.6	
	IL-6	5E+1	112.0 ± 3.6	99.7 ± 7.0 %
		5E+2	82.8 ± 5.0	
		5E+3	85.6 ± 4.8	
		1E+6	104.9 ± 12.3	
		1E+7	100.1 ± 7.6	
		1E+8	112.9 ± 9.0	
	IFN-γ	5E+1	118.7 ± 13.5	108.5 ± 6.5 %
		5E+2	108.1 ± 4.4	
		5E+3	98.8 ± 1.6	
	CRP	1E+6	94.5 ± 3.1	94.6 ± 4.5 %
		1E+7	99.3 ± 3.7	
		1E+8	90.1 ± 6.6	

9 Method Comparison

9.1 Individual Biomarker Comparison

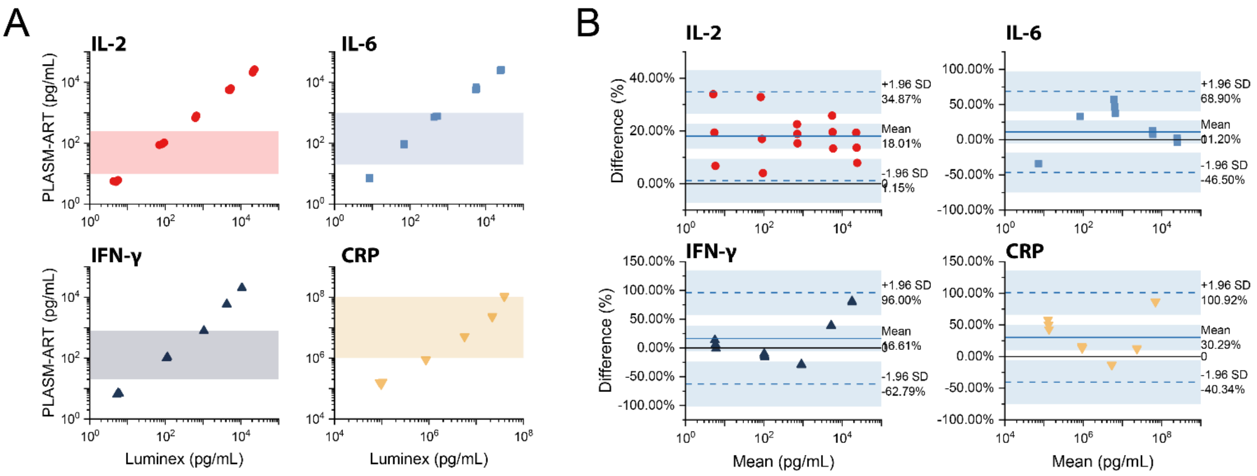

Figure S12. (A) Scatter plots show the quantitative agreement between the PLASM-ART platform and Luminex assay for IL-2, IL-6, IFN-γ, and CRP. Shaded regions denote the clinically relevant dynamic range for each marker. (B) Bland-Altman analysis comparing Luminex measurements to known spiked concentrations for each analyte. Solid lines indicate the mean bias; dashed lines represent ±1.96 standard deviation (SD) limits.

Table S3. Summary on each protein, including source and associated antibodies (capture/detection, specificity, and isotype).

Protein	IL-2	IL-6	IFN-γ	CRP
**Protein Source**	Source: Mabtech<br>Type: Recombinant<br>Reactivity: Human	Source: HyTest<br>Type: Recombinant<br>Reactivity: Human	Source: Mabtech<br>Type: Recombinant<br>Reactivity: Human	Source: HyTest<br>Type: Recombinant<br>Reactivity: Human
**Capture Antibody**	Source: Mabtech<br>Cat#: MT2A91/2C95<br>Isotype: IgG2b/IgG1<br>Origin: Mouse<br>Reactivity: Human	Source: HyTest<br>Cat#: L519<br>Isotype: IgG1<br>Origin: Rat-human<br>Reactivity: Human	Source: Mabtech<br>Cat#: 1-D1K<br>Isotype: IgG1<br>Origin: Mouse<br>Reactivity: Human	Source: HyTest<br>Cat#: C2cc<br>Isotype: IgG2a<br>Origin: Mouse<br>Reactivity: Human
**Detection Antibody**	Source: Mabtech<br>Cat#: MT8G10<br>Isotype: IgG1<br>Origin: Mouse<br>Reactivity: Human	Source: HyTest<br>Cat#: L395<br>Isotype: IgG1<br>Origin: Rabbit<br>Reactivity: Human	Source: Mabtech<br>Cat#: 7-B6-1<br>Isotype: IgG1<br>Origin: Mouse<br>Reactivity: Human	Source: HyTest<br>Cat#: C6cc<br>Isotype: IgG2a<br>Origin: Mouse<br>Reactivity: Human
**Notes**	Not applicable	**L519:** Chimeric rat-human IgG1 antibody with rat variable and human constant domains, expressed in mammalian cells.<br><br>**L395:** Full-length recombinant rabbit antibody expressed in mammalian cells.	Not applicable	Not applicable

10 Automated Image Analysis

10.1 Analysis

To accurately quantify target concentrations across a broad dynamic range, we developed a hybrid digital-analog response metric. At low analyte levels, individual AuNPs are well-separated and readily segmented, enabling discrete particle counting with single-event resolution. However, as target concentration increases, particle density within the capture spots rises sharply, leading to signal saturation, spatial overlap, and intensity gradients that hinder reliable segmentation. In this high-density regime, purely digital counting underestimates the true analyte abundance due to unresolvable clustering. To overcome this limitation while retaining digital sensitivity at the low end, we devised a composite metric that adaptively integrates particle count, optical contrast, and signal heterogeneity. This formulation enables quantification across both sparse and crowded conditions, enhancing the sensor's dynamic range without sacrificing the resolution of single events in the digital regime. Below, we describe the mathematical structure of this adaptive response metric.

The metric is defined as:

$$Response = \left(\frac{Spot\ NPs}{A/_{A_{ref}}} \right)^{1-CR} \times m_2 \qquad \text{Eq. S20}$$

where Spot NPs is the number of segmented AuNPs within the spot region, A is the spot area, A_{ref} is a normalization constant corresponding to a typical spot size. The first term captures particle density, normalized by area; the exponent dynamically adjusts its weight based on contrast. High-contrast spots diminish the influence of particle count, while low-contrast spots restore it. This adaptive scaling ensures that contrast and particle abundance are considered jointly, without overemphasizing either in extreme cases. The final multiplicative factor, m_2, captures intra-spot signal dispersion by quantifying the second moment of the histogram of individual particle contrast ratios. This term favors spots with a balanced distribution of particle intensities and penalizes outlier-dominated spots, adding a layer of quality control beyond total signal strength.

The contrast ratio CR serves as an exponent to modulate the influence of particle count based on optical clarity, and defined as,

$$CR = \frac{C_{spot} - C_{bg}}{(255 - C_{bg}).\frac{\overline{C_{bg}}}{C_{bg}}} \qquad \text{Eq. S21}$$

where C_{spot} and C_{bg} are the inverted average grayscale intensities of the spot and its local background, respectively, and $\overline{C_{bg}}$ is the mean background across all spots.

The second moment m_2 quantifies the spread of particle-level contrast ratios within a spot. It is defined as,

$$m_2 = \frac{\sum_i c_i^2 h_i}{\sum_i c_i h_i} \qquad \text{Eq. S22}$$

where c_i is the center of the i-th bin of the contrast histogram and h_i is the corresponding count. This term captures the distribution of particle intensities within the spot. Since particle brightness scales with the number of particles in close proximity, elevated m_2 values indicate signal amplification due to particle clustering. By incorporating this measure, response is sensitive not only to particle number and contrast, but also to sub-spot spatial organization that enhances detection.

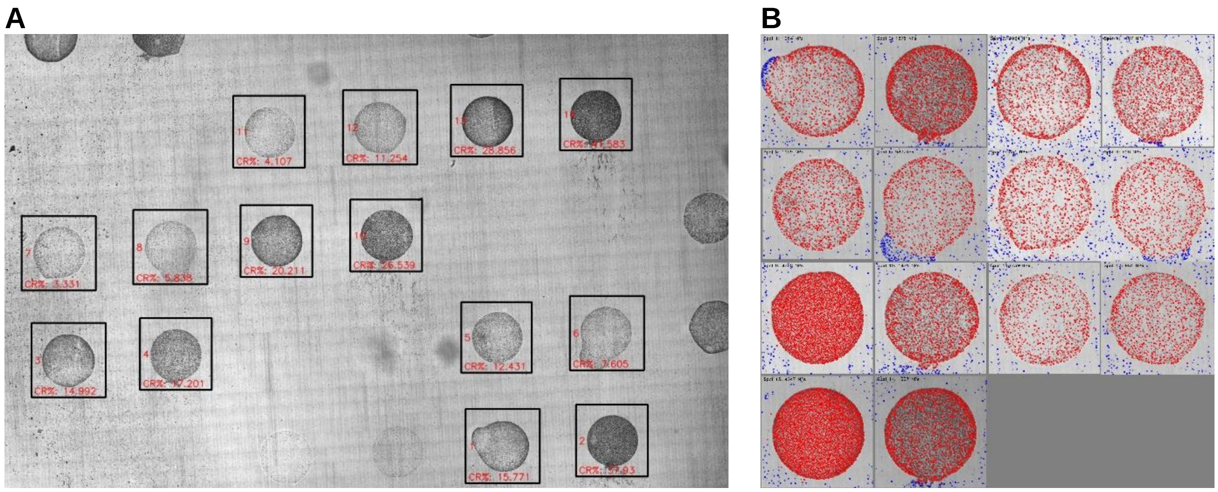

Figure S13. Automated image analysis of spot detection and AuNP segmentation. (A) Detected antibody spots on a representative chip image, outlined with black bounding boxes. Each spot is annotated with a contrast ratio (CR%) value, quantifying the relative brightness difference between the spot center and surrounding background. **(B)** Binary segmentation masks generated from high-magnification scans of individual spots. Red pixels denote classified AuNPs, while blue pixels indicate artifacts or noise rejected during filtering. This segmentation enables particle-level quantification and spatial pattern analysis across spots, facilitating consistent and objective response extraction.

10.2 Flowchart

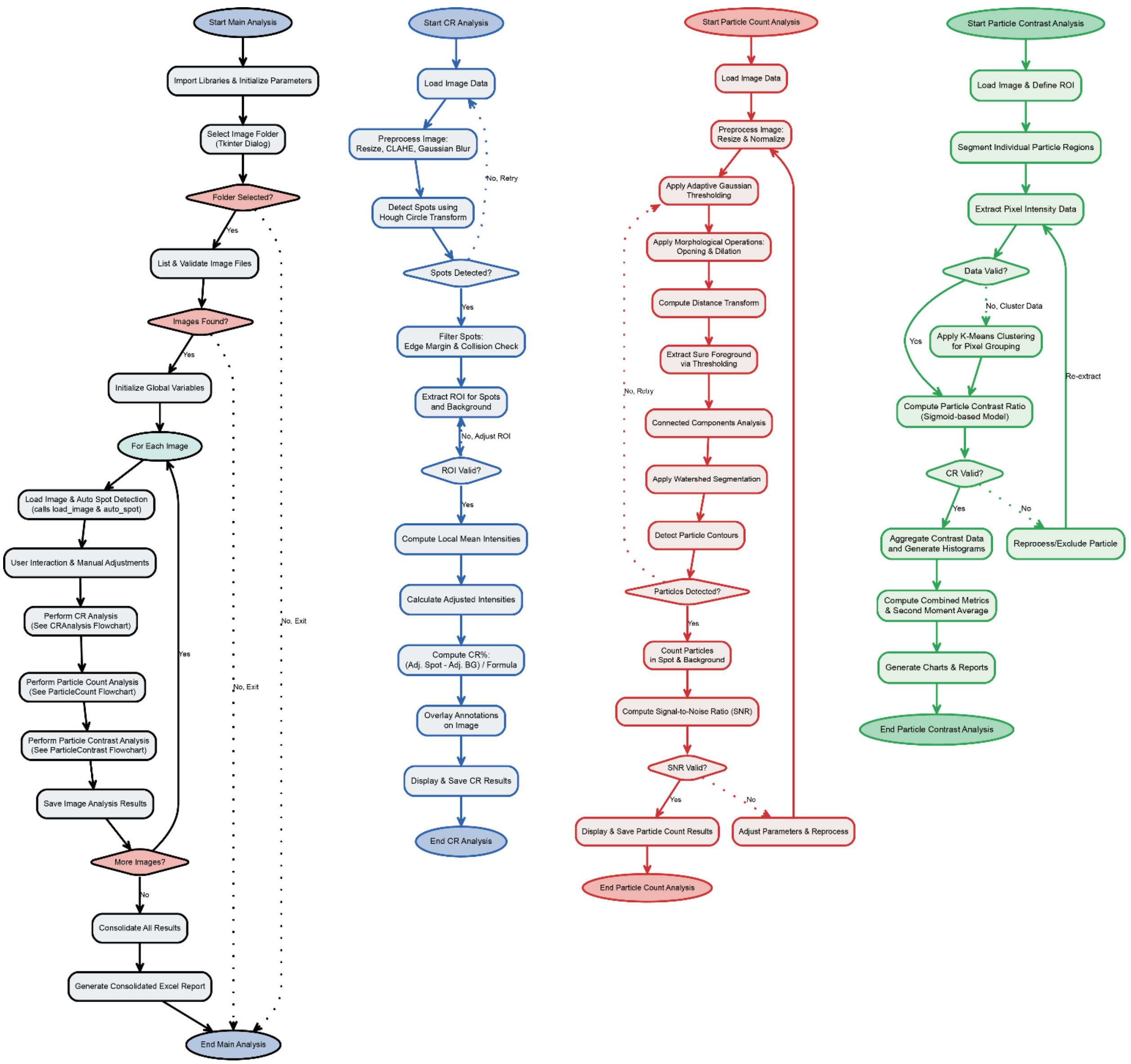

Figure S14. Flowchart of image analysis pipeline for multiplexed biosensing. The diagram outlines the complete computational workflow used to analyze biosensor images, composed of four interconnected modules: Main Analysis (black), CR (Contrast Ratio) Analysis (blue), Particle Count Analysis (red), and Particle Contrast Analysis (green). The Main Analysis module initializes parameters, loads image files, and orchestrates downstream analyses. The CR Analysis module detects assay spots via Hough Circle Transform, computes adjusted local intensities, and calculates the CR metric. The Particle Count Analysis module performs adaptive thresholding, segmentation, and connected component analysis to quantify particle number and signal-to-noise ratio (SNR). The Particle Contrast Analysis module extracts per-particle intensity profiles, applies k-means clustering to validate regions, and computes second moment contrast metrics. Conditional loops enable reprocessing of invalid data and user adjustments where needed. Final

outputs include visual annotations, particle statistics, and consolidated reports for quantitative comparison across experimental conditions.

REFERENCES

(1) Armbruster, D. A.; Pry, T. Limit of Blank, Limit of Detection and Limit of Quantitation. *Clin Biochem Rev* **2008**, *29 Suppl 1* (Suppl 1), S49-52.

(2) Shoup, D.; Lipari, G.; Szabo, A. Diffusion-Controlled Bimolecular Reaction Rates. The Effect of Rotational Diffusion and Orientation Constraints. *Biophysical Journal* **1981**, *36* (3), 697–714. https://doi.org/10.1016/s0006-3495(81)84759-5.

(3) Shoup, D.; Szabo, A. Role of Diffusion in Ligand Binding to Macromolecules and Cell-Bound Receptors. *Biophysical Journal* **1982**, *40* (1), 33–39. https://doi.org/10.1016/s0006-3495(82)84455-x.

(4) Karlsson, R.; Roos, H.; Fägerstam, L.; Persson, B. Kinetic and Concentration Analysis Using BIA Technology. *Methods* **1994**, *6* (2), 99–110. https://doi.org/10.1006/meth.1994.1013.

(5) Jennissen, H. P.; Zumbrink, T. A Novel Nanolayer Biosensor Principle. *Biosensors and Bioelectronics* **2004**, *19* (9), 987–997. https://doi.org/10.1016/j.bios.2003.09.008.

(6) Jennissen, H. P. Boundary-Layer Exchange by Bubble: A Novel Method for Generating Transient Nanofluidic Layers. *Physics of Fluids* **2005**, *17* (10), 100616. https://doi.org/10.1063/1.1990207.

(7) Daniel, C.; Roupioz, Y.; Gasparutto, D.; Livache, T.; Buhot, A. Solution-Phase vs Surface-Phase Aptamer-Protein Affinity from a Label-Free Kinetic Biosensor. *PLoS ONE* **2013**, *8* (9), e75419. https://doi.org/10.1371/journal.pone.0075419.

(8) Ward, G.; Mckinnon, L.; Badrick, T.; Hickman, P. E. Heterophilic Antibodies Remain a Problem for the Immunoassay Laboratory. *Am J Clin Pathol* **1997**, *108* (4), 417–421. https://doi.org/10.1093/ajcp/108.4.417.

(9) Kricka, L. J. Human Anti-Animal Antibody Interferences in Immunological Assays. *Clinical Chemistry* **1999**, *45* (7), 942–956. https://doi.org/10.1093/clinchem/45.7.942.

(10) Tate, J.; Ward, G. Interferences in Immunoassay. *Clin Biochem Rev* **2004**, *25* (2), 105–120.

(11) Howanitz, J. H. *Immunoassay Interference by Endogenous Antibodies: Approved Guideline*; Clinical and Laboratory Standards Institute : IFCC: Wayne, Pa., 2008.

(12) Levinson, S. S.; Miller, J. J. Towards a Better Understanding of Heterophile (and the like) Antibody Interference with Modern Immunoassays.

(13) Ismail, A.; Walker, P.; Cawood, M.; Barth, J. Interference in Immunoassay Is an Underestimated Problem. *Ann Clin Biochem* **2002**, *39* (4), 366–373. https://doi.org/10.1258/0004563021902128.

(14) Bolstad, N.; Warren, D. J.; Nustad, K. Heterophilic Antibody Interference in Immunometric Assays. *Best Practice & Research Clinical Endocrinology & Metabolism* **2013**, *27* (5), 647–661. https://doi.org/10.1016/j.beem.2013.05.011.

(15) Hennig, C.; Rink, L.; Fagin, U.; Jabs, W. J.; Kirchner, H. The Influence of Naturally Occurring Heterophilic Anti-Immunoglobulin Antibodies on Direct Measurement of Serum Proteins Using Sandwich ELISAs. *Journal of Immunological Methods* **2000**, *235* (1–2), 71–80. https://doi.org/10.1016/s0022-1759(99)00206-9.

(16) Boscato, L. M.; Stuart, M. C. Heterophilic Antibodies: A Problem for All Immunoassays. *Clinical Chemistry* **1988**, *34* (1), 27–33. https://doi.org/10.1093/clinchem/34.1.27.

(17) Mohammadi, M. M.; Bozorgi, S. Investigating the Presence of Human Anti-Mouse Antibodies (HAMA) in the Blood of Laboratory Animal Care Workers. *Journal of Laboratory Medicine* **2019**, *43* (2), 87–91. https://doi.org/10.1515/labmed-2018-0084.

(18) Survey of Methods for Measuring Human Anti-Mouse Antibodies. *Clinica Chimica Acta* **1993**, *215* (2), 153–163. https://doi.org/10.1016/0009-8981(93)90122-k.

(19) Dekkers, G.; Bentlage, A. E. H.; Stegmann, T. C.; Howie, H. L.; Lissenberg-Thunnissen, S.; Zimring, J.; Rispens, T.; Vidarsson, G. Affinity of Human IgG Subclasses to Mouse Fc

Gamma Receptors. *mAbs* **2017**, *9* (5), 767–773. https://doi.org/10.1080/19420862.2017.1323159.

(20) Kragstrup, T. W.; Vorup-Jensen, T.; Deleuran, B.; Hvid, M. A Simple Set of Validation Steps Identifies and Removes False Results in a Sandwich Enzyme-Linked Immunosorbent Assay Caused by Anti-Animal IgG Antibodies in Plasma from Arthritis Patients. *SpringerPlus* **2013**, *2* (1). https://doi.org/10.1186/2193-1801-2-263.
